# Alcohol Exposure May Increase Prenatal Choline Needs Through Redirection of Choline into Lipid Synthesis Rather than Methyl Donation

**DOI:** 10.3390/metabo15050289

**Published:** 2025-04-24

**Authors:** Hannah G. Petry, Nipun Saini, Susan M. Smith, Sandra M. Mooney

**Affiliations:** 1 Department of Nutrition, University of North Carolina, Chapel Hill, NC 27599, USA; hannah_petry@unc.edu (H.G.P.); nipun_saini@unc.edu (N.S.); 2 UNC Nutrition Research Institute, Kannapolis, NC 28081, USA

**Keywords:** fetal alcohol spectrum disorder, Kennedy pathway, metabolome, mouse, one carbon metabolism, prenatal alcohol

## Abstract

**Background**: Prenatal alcohol exposure (PAE) can reduce fetal growth and cause neurodevelopmental disability. Prenatal choline supplements attenuate PAE-induced behavioral and growth deficits; however, the underlying mechanisms are unknown. Alcohol alters nutrient metabolism and potentially increases nutrient needs. Here, we investigate how alcohol affects choline metabolism in the maternal–fetal dyad and the role of supplemental choline. **Methods**: Pregnant C57BL/6J mice were assigned to one of four groups: alcohol-exposed (3 g/kg alcohol/day) or control +/− 100 mg/kg choline daily from embryonic day (E)8.5–17.5. We performed an exploratory hypothesis-generating analysis of targeted metabolomics on choline-related metabolites in the maternal liver, plasma, placenta, and fetal brain at E17.5 and Spearman correlation analyses to determine their association with gestational and fetal growth outcomes. **Results**: Although choline levels were largely unaffected by alcohol or choline, alcohol increased many lipid products in the CDP–choline pathway; this was not normalized by choline. Alcohol increased placental CDP–ethanolamine and reduced the maternal hepatic SAM/SAH ratio as well as dimethylglycine and the serine/glycine ratio across the dyad, suggesting a functional insufficiency in methyl donor pools. These outcomes were rescued by supplemental choline. Correlation analyses among choline metabolites and fetal growth outcomes suggest that maternal plasma methionine, serine, and the serine/glycine ratio may be predictive of maternal–fetal choline status. **Conclusions**: The increased hepatic lipid synthesis that characterizes chronic alcohol exposure may draw choline into phospholipid biosynthesis at the expense of its use as a methyl donor. We propose that PAE increases choline needs, and that its supplementation is necessary to fulfill these competing demands for lipid and methyl use.

## 1. Introduction

Fetal alcohol spectrum disorder (FASD), which can result from prenatal alcohol exposure (PAE), is a leading cause of neurobehavioral deficits affecting domains including attention, cognition, learning, memory, and executive function [1,2,3,4]. In the USA, screening studies find that 2–5% of first-grade children meet the diagnostic criteria for FASD [5], an incidence that aligns with self-reports that 13.5% of pregnant adults in the USA consumed alcohol and 5.2% binge drank in the previous 30 days [6]. The behavioral deficits of FASD persist into adulthood, and there is great interest in interventions that mitigate alcohol’s prenatal damage.

One such intervention is the essential nutrient choline. Preclinical studies document that supplemental choline, whether administered prenatally or postnatally, improves the offspring’s PAE-induced growth and cognitive impairments [7,8,9]. Clinical studies concur; infants born to heavy-drinking women who received supplemental choline plus multivitamins/minerals had improved visual memory in a habituation–dishabituation test [10]. In a separate cohort, maternal adherence to a 2 g/day prenatal choline intervention was linearly related to their infant’s regional brain volume and performance in an eyeblink conditioning task [11,12]. Young children (aged 2.5–5 years) with FASD showed greater pre/post improvements in an elicited imitation memory task following a 9-month choline intervention [13]. These benefits persisted, and those who received choline had better performance in measures of non-verbal IQ, working memory, and spatial reasoning at the 4-year follow-up [14] and in executive function and white matter microstructure at the 7-year follow-up [15]. Although choline has been shown to reverse alcohol’s effects with respect to, for example, cell death [16], process outgrowth [17], microRNA expression [18], and DNA methylation [19,20,21,22], the underlying mechanisms by which choline achieves these benefits remain poorly understood. Moreover, it is also unclear whether alcohol interacts with choline to increase maternal–fetal choline needs or if it directly affects choline metabolism.

Insights emerge from studies that show that choline is essential for healthy brain development as it contributes to the major brain lipids, phosphatidylcholine and sphingomyelin, and the neurotransmitter acetylcholine [23,24,25]. It is also the primary source of methyl groups for over 100 biochemical reactions, including the synthesis of purines, pyrimidines, select amino acids, heme, and DNA and histone methylation. Prenatal choline insufficiency is associated with behavioral impairments similar to those of FASD, including attention, learning, and memory, whereas its supplementation improves those measures [24,26,27]. Unfortunately, only ~10% of pregnant women in the US consume sufficient choline to meet the adequate intake (AI) of 450–480 mg/day, suggesting that most pregnancies are choline-insufficient [28,29].

**Figure 1:**
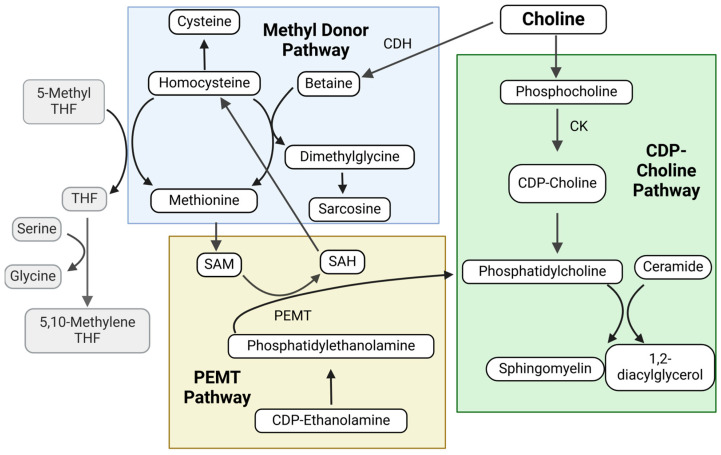
Cellular choline metabolism pathways. CDH—choline dehydrogenase; CDP—cytidine diphosphate; CK—choline kinase; PEMT—Phosphatidylethanolamine N-Methyltransferase; SAH—S-Adenosyl Homocysteine; SAM—S-Adenosyl Methionine; THF—tetrahydrofolate. Created in BioRender. Petry, H. (https://www.biorender.com/, accessed on 25 February 2025).

Three major pathways govern choline’s cellular fate: the phosphatidylethanolamine methyltransferase (PEMT) pathway, the cytidine diphosphate–choline (CDP-choline, also known as citicoline) pathway, and the methyl donor pathway. In the first pathway, choline is generated endogenously by the liver via the PEMT-mediated transfer of methyl groups from methionine (as S-adenosylmethionine (SAM)) to phosphatidylethanolamine to make phosphatidylcholine (Figure 1). However, PEMT activity generates just ~30% of hepatic phosphatidylcholine and thus is insufficient to meet needs [27,30]. This is especially true during pregnancy, when the high anabolic demands deplete maternal liver choline [31]. Thus, dietary choline is necessary to fully meet these needs. Some dietary choline is directly incorporated into phosphatidylcholine to support cell membrane structure via the CDP–choline (or Kennedy) pathway. Exogenous choline also supports methyl donation reactions (methyl donor pathway) through its mitochondrial oxidation to betaine, which then donates its methyl groups to homocysteine to yield methionine and dimethylglycine. This methionine can then be used to produce the final methyl donor SAM [27,32]. Studies suggest that the methyl donor pathway is adversely affected by choline insufficiency. In a choline-deficient pregnancy, choline supplementation increases choline, betaine, and dimethylglycine in the maternal liver [33] and choline, phosphatidylcholine, and betaine in the maternal plasma [34]. A tracer study in pregnant rats showed that dietary labeled choline mostly appears as phosphatidylcholine and phosphocholine in the fetus [34]. Similar tracer studies performed in the third trimester in women who were otherwise choline adequate showed that this choline preferentially entered the CDP–choline and PEMT pathways to produce phosphatidylcholine over betaine synthesis [35,36].

To generate insights into how alcohol might interact with supplemental choline to affect choline metabolism, utilization, and needs in the maternal–fetal dyad, we performed a targeted metabolomic analysis of choline-related metabolites in a mouse model of FASD. This model has parallels with alcohol-related neurodevelopmental disorder (ARND) with deficits in memory, learning, and fetal brain weight, yet normal body weight and survival [37,38]. We find that, in the late term, PAE is associated with elevations of phosphatidylcholine and other lipid intermediates at the expense of metabolites in the methyl donor pathway and that supplemental choline, administered to the mother at a dose that normalizes offspring growth and behavior [8,37], restores this balance.

## 2. Materials and Methods

### 2.1. Animals and Diet

Pair-housed five-week-old female C57BL/6J mice (Jackson Laboratories, Bar Harbor, ME, USA) consumed a nutritionally adequate defined diet (AIN-93G; TD.94045; Envigo Teklad, Madison, WI, USA) [39] throughout this study. At eight weeks of age, they were bred overnight with C57BL/6J males. The morning a vaginal plug was observed was designated as embryonic day (E) 0.5. On E 8.5, dams were assigned to one of the four experimental groups using a random-number generator: control (CON), alcohol-exposed (ALC), control + choline-treated (CON-Cho), or alcohol-exposed + choline-treated (ALC-Cho; N = 8 for all groups). Mice received a once-daily oral gavage of 3 g/kg of ethanol (200 proof alcohol, USP grade; Decon Labs, King of Prussia, PA, USA) or isocaloric maltodextrin as control (LoDex-10; #160175, Envigo Teklad, Madison, WI, USA). Choline-treated (Cho) dams received a subcutaneous injection of 100 mg/kg of choline chloride (#F6522120; Balchem, New Hampton, NY, USA) [8,33] once daily immediately after oral gavage (E8.5–E17.5). This choline injection provides 74.6 mg of choline/kg body weight and equates to 2.4 mg of choline/day for a dam weighing 32 g. AIN-93G contains 1 mg choline/g diet [39] and these dams consumed an average of 3.2 mg of choline/day; thus, Cho mice received 5.6 mg of choline/day, and this represents 75% of additional choline/day or 175% of the mouse AI, which is comparable to 175% of the choline AI for humans.

On E 17.5, four hours after gavage, dams were euthanized by isoflurane overdose. Maternal and fetal tissues were weighed and flash frozen for analysis. Maternal plasma and liver samples consisted of 8 biological replicates per group. Placenta and fetal brain samples consisted of a pool of 4 fetuses taken from each of the 8 dams, and most samples contained 2 male and 2 female fetuses. Fetal weights are the average for all fetuses in a litter. Placental efficiency was calculated as fetal body weight divided by the placenta weight for each fetus and presented as average placental efficiency per litter. All procedures were approved by the Institutional Animal Care and Use Committee (IACUC) of the North Carolina Research Campus.

### 2.2. Metabolomics and Data Analysis

Tissue samples were sent to Metabolon (Durham, NC, USA) for untargeted metabolite analysis using ultra-high-performance liquid chromatography–mass spectrometry (UPLC-MS); the details of the metabolite separation, detection, and alignment performed at Metabolon are presented elsewhere [40]. In brief, protein was extracted from the samples using methanol, and then each sample was split into five aliquots. These were subjected to reverse phase (RP) UPLC-MS/MS with positive ion mode electrospray ionization (ESI, 2 aliquots), RP/UPLC-MS/MS with negative mode ESI (one aliquot), and HILIC/UPLC-MS/MS with negative ion mode ESI (one aliquot). The fifth aliquot was reserved as a back-up. The sampling order was randomized, and the controls included water and solvent blanks, technical replicates of pooled samples, recovery standards to assess efficiency and variability, and internal standards to assess instrument variability and aid in chromatographic alignment. Identities were based on retention time/index, a match to the mass-to-charge ratio ± 10ppm, and the MS/MS spectrum using proprietary software in 2019 [40] Individual metabolites were quantified using the area under the curve (AUC) of each peak. We excluded samples with a total abundance for all metabolites that was more than two standard deviations below the mean abundance for all samples. N = 8 for all groups except for plasma, where CON and ALC N = 8, CON-Cho N = 6, and ALC-Cho N = 7. Individual metabolites that were not detected in at least 5 samples per group were also excluded from the analysis. For metabolites not detected in 3 or fewer samples, values were imputed using the minimum detectable value for that metabolite [40]. From this dataset, choline-related metabolites, as defined elsewhere [41], were extracted for targeted analysis (Supplemental Table S1). The lipid classes of phosphatidylcholines, phosphatidylethanolamines, sphingomyelins, diacylglycerols, and ceramides each represent a mixture that features different fatty acyl moieties, and their mean values are the summed abundance of the individual metabolite species within that class (Supplemental Table S2).

Data were tested for normality and equal variance using Levene’s and Shapiro–Wilks tests, respectively. Most metabolites did not have a normal distribution; therefore, nonparametric Mann–Whitney U-tests (Wilcoxon Rank sum tests) were used to identify fold-change differences (FC) between pairs of groups (ALC vs. CON; ALC-Cho vs. ALC; CON-Cho vs. CON; ALC-Cho vs. CON). Data for metabolite classes and for the SAM/SAH and serine/glycine ratios met normality; therefore, parametric unpaired *t*-tests were conducted to identify the FC between these groups. For all tests, *p*-values were adjusted for multiple comparisons using the Benjamini–Hochberg False Discovery Rate (FDR). Significance was determined as FDR < 0.05 and trending as 0.05 < FDR < 0.10. Because the tissue levels of many metabolites are normally under tight regulation, the effect sizes were calculated for metabolites when the group mean differences exceeded 10% or when differences were statistical trends using the rank-biserial correlation coefficient (r) for nonparametric data or Cohen’s d for parametric data [42]. For the rank-biserial correlation coefficient, r < 0.3 is small, 0.3 < r < 0.5 is medium, and r > 0.5 is a large effect. For Cohen’s d, d = 0.2 is small, d = 0.5 is medium, and d = 0.8 is a large effect. Outcomes are focused on findings that were significant and where a medium or large effect size was identified.

Spearman’s rank correlation coefficient was used to determine correlations among choline-related metabolites and pregnancy and birth outcomes, with significance regarded at *p* < 0.05. Correlation coefficients below 0.3 are weak, between 0.4 and 0.6 are moderate, and between 0.7 and 1 are strong. Heatmaps were created using ggplot2 (version 3.5.1) and dplyr (version 1.1.4) packages to visualize Spearman correlation analyses. Boxplots were created using the ggplot2 (version 3.5.1) package with abundance relative to CON. Statistical analyses were performed using R-Studio (version 4.4.2) and GraphPad Prism (version 10).

## 3. Results

### 3.1. Alcohol Alters the Abundance of Choline-Related Metabolites in the Maternal–Fetal Dyad

The untargeted metabolite analysis detected 779, 814, 866, and 615 metabolites in the maternal plasma, maternal liver, placenta, and fetal brain, respectively. Three plasma samples (two from CON-Cho and one from ALC-Cho) had a total abundance from all 779 metabolites that exceeded two standard deviations below the mean, raising concerns about their collection or preparation, and these were excluded from further analysis. The sample sizes were N = 8 for all groups except for plasma, where CON and ALC N = 8, CON-Cho N = 6, and ALC-Cho N = 7. We identified 90, 102, 100, and 77 choline-related metabolites in the remaining samples from maternal plasma, maternal liver, placenta, and the fetal brain, respectively (Supplemental Table S1); most were detected across all tissues, with the exception of CDP–choline (plasma), CDP–ethanolamine (plasma), SAM (plasma), and sarcosine (plasma; placenta). We detected 19–22 distinct phosphatidylcholines, 11–13 phosphatidylethanolamines, 15–28 sphingomyelins, 4–9 ceramides, and 9–25 diacylglycerols (Supplemental Table S2); their presence was a function of the tissue and not the experimental group. The FCs of abundance of choline-related metabolites in all four tissues are presented for the comparisons of ALC vs. CON (Table 1), ALC-Cho vs. ALC (Table 2), ALC-Cho vs. CON (Supplemental Table S3), and CON-Cho vs. CON (Supplemental Table S4). We also assessed 5-methyltetrahydrofolate (5MeTHF) for insights into the folate pathway; this was the only folate metabolite detected (Supplemental Figure S1).

#### 3.1.1. Choline

Choline levels in maternal plasma, placenta, and the fetal brain were largely unaffected by alcohol exposure or choline supplementation, and this was expected as it is tightly regulated [43]. The exception was liver, which is a primary site of choline metabolism. Although maternal hepatic choline was 25% higher in ALC vs. CON (Table 1) and 10% higher in the ALC dams receiving choline (Table 2), these were small effects; however, they combined in the ALC-CHO vs. CON comparison to significantly increase choline by 38% (FDR < 0.05; Supplemental Table S3). Choline supplementation in CON dams increased their hepatic choline content by only 9% (Supplemental Table S4). In summary, alcohol exposure did not cause a choline deficiency per se and instead increased its hepatic content.

#### 3.1.2. CDP–Choline Pathway

This pathway directly incorporates choline into the synthesis of phosphatidylcholine and the specialized lipids ceramide and sphingomyelin. In general, alcohol exposure increased the abundance of the products synthesized by this pathway compared with CON (Table 1). This was evidenced in maternal compartments, where alcohol increased the total phosphatidylcholine (119% of CON), ceramide (139% of CON), and sphingomyelin (128% of CON) content in maternal plasma, ceramide (143% of CON), and sphingomyelin (112% of CON) in maternal liver and phosphatidylcholine (114% of CON) and sphingomyelin (111% of CON) content in placenta. In contrast, these were unaffected by alcohol in the fetal brain. Choline supplementation did not further affect these lipid levels in the ALC dams and fetuses (Table 2). With respect to the starting substrates, alcohol non-significantly increased phosphocholine in the placenta (113% of CON) and showed a small effect on phosphocholine in the maternal liver (122% of CON) that was magnified by supplemental choline (140% of ALC, 171% of CON). Similarly, alcohol elevated CDP–choline in the fetal brain (123% of CON) (Table 1), and this was reversed by choline (66% of ALC, 81% of CON) (Table 2 and Supplemental Table S3). In contrast, in CON mice, supplemental choline increased phosphocholine and CDP–choline only in the maternal liver (165% and 150% of CON, respectively) (Supplemental Table S4). In summary, alcohol increased the abundance of CDP–choline pathway substrates in the placenta and fetal brain and products in the maternal and placental tissues; some substrates were further elevated by supplemental choline and may reflect that choline kinase is not subject to allosteric regulation [44].

#### 3.1.3. PEMT Pathway

The PEMT pathway synthesizes de novo choline as phosphatidylcholine from SAM and CDP–ethanolamine (Figure 1). Alcohol increased the substrates CDP-ethanolamine in placenta (129%) and phosphatidylethanolamine in placenta (109%) and liver (129%) (Table 1). Choline supplementation reversed alcohol’s effects on CDP–ethanolamine but not on phosphatidylethanolamine (Table 2). Its effect in the controls was different, and choline supplementation increased hepatic CDP–ethanolamine (119% of CON) and placental phosphatidylethanolamine (110%) (Supplemental Table S4). In summary, alcohol exposure increased the availability of ethanolamine substrates in the PEMT pathway, and this was partly attenuated by choline.

SAM donates the methyl group for PEMT activity, whereas its product SAH inhibits the methyltransferase; thus, the SAM/SAH ratio informs the potential flux through the PEMT pathway and for other methyltransferases including those targeting DNA. ALC reduced the hepatic SAM content (73% of CON) and increased the placental SAH (119% of CON) (Table 1). These led to reduced SAM/SAH ratios in both the maternal liver (72% of CON) and placenta (85% of CON) by alcohol, which would favor reduced methyltransferase activity in those tissues. Choline supplementation raised hepatic SAM (126% of ALC), SAH (112% of ALC), and the SAM/SAH ratio (115% of ALC) and reduced plasma SAH (64% of ALC; Table 2); however, hepatic and placental SAM/SAH were still below CON values (83% and 88%, respectively; Supplemental Table S3). With respect to fetal brain, ALC modestly increased the SAM/SAH ratio (112% of CON), and choline slightly reduced it in ALC (94%), thereby returning it to levels seen in CON, suggesting that ALC effects on SAM/SAH were greatest in the dam and placenta. Supplemental choline did not affect the SAM/SAH ratio in CON maternal tissues or placenta, perhaps reflecting their tight control under normal conditions and suggesting their dysregulation by ALC, but it decreased SAH (86% of CON) and increased the SAM/SAH ratio (115% of CON) in the fetal brain.

#### 3.1.4. Methyl Donor Pathway

The methyl groups on SAM are obtained via their transfer from free choline in the form of betaine to generate methionine and dimethylglycine; dimethylglycine is further metabolized to sarcosine, which then generates glycine and the methyl donor methylene-tetrahydrofolate (THF) when serine is limiting. Although alcohol did not affect betaine levels, it reduced hepatic dimethylglycine (71% of CON) (Table 1). Methionine levels, which are normally under tight regulation, were consistently less than 90% of CON in both maternal and fetal tissues. In contrast, cysteine, which is generated from homocysteine (i.e., demethylated methionine) via the transsulfuration of serine, was elevated by alcohol in the maternal plasma (112%) and the fetal brain (138%). These differences were largely reversed by supplemental choline, which increased hepatic betaine (154%), dimethylglycine (170%), and sarcosine (172%) above the levels seen in ALC (Table 2) and even above those in CON (121%—160%; Supplemental Table S3). Although supplemental choline also elevated these in CON liver, its impact was attenuated (range from 114% to 144%) (Supplemental Table S4). In contrast, choline did not normalize methionine levels in ALC tissues and, in CON, it reduced methionine (74–86%) while increasing cysteine in all but the maternal liver. Overall, ALC reduced metabolites within this pathway, and choline raised them to a greater extent than in CON.

#### 3.1.5. Other Metabolites

Although many metabolites are produced from SAM-donated methyl groups, we focus here on those directly relevant for choline status. Two important ones are the amino acids serine and glycine; methyl transfer from serine to THF generates glycine and 5,10-methylene–THF for additional methyl donor reactions. Stressors that reduce serine increase the need to regenerate methionine and SAM from choline [45,46,47] while converting the dimethylglycine into glycine to meet the latter needs. Thus, the serine/glycine ratio informs the additional demands for choline-donated methyl groups. Alcohol decreased the serine/glycine ratio in all tissues, including the placenta (81%) and the fetal brain (86%), and this was driven largely by reductions in serine (Table 1). Supplemental choline improved serine content and the serine/glycine ratios in the maternal plasma, liver, and placenta (Table 2); however, for the fetal brain, both serine and the serine/glycine ratio remained lower compared with CON (Supplemental Table S3). In CON, supplemental choline had little effect on these metabolites or their ratios.

To provide more insight into the folate pathway, we assessed its only identified metabolite, 5MeTHF. Alcohol reduced 5MeTHF in the placenta (78% of CON), and supplemental choline increased this (130% of ALC) (Supplemental Figure S1). In CON mice, supplemental choline decreased 5MeTHF (54% of CON) in the maternal liver.

Choline that enters the colon can be metabolized by the microbiota to trimethylamine, which is oxidized by the liver to trimethylamine N-oxide (TMAO). Alcohol reduced TMAO in all tissues, with the strongest effects in the maternal plasma (41% of CON) and liver (36%; Table 1). Although choline supplementation in ALC dams selectively elevated tissue TMAO by 128% to 244% in maternal and fetal tissues (Table 2), these values did not exceed those in the CON pregnancies (range of 100% to 88%) (Supplemental Table S3). Choline did not elevate TMAO in the control pregnancies, and decreased levels in the maternal liver (59% of CON) (Supplemental Table S4). In summary, prenatal alcohol exposure was associated with increased hepatic choline, increased CDP–choline and PEMT pathway intermediates, and reduced methyl donor intermediates and SAM/SAH ratios, mostly affecting the mother and placenta. Choline supplementation in the ALC pregnancies further elevated the abundance of choline and CDP–choline intermediates and normalized the SAM/SAH ratios and PEMT and methyl donor pathway intermediates, again mostly in the mother and placenta.

### 3.2. Plasma Choline-Related Metabolites Correlate with Pregnancy and Fetal Outcomes

To gain insights into how these differences in choline metabolism impact gestational and fetal growth outcomes, we conducted Spearman correlation analyses comparing the abundance of choline-related metabolites to gestational weight gain, placental weight and efficiency, and fetal weights (Table 3 and Supplemental Figure S2). Most of these correlations were in the small-to-moderate range. A subset of these weights was previously reported [37], and a list of the mean weight outcomes for each group can be found in Supplemental Table S5. We focused initially on maternal plasma given its clinical utility. Plasma choline itself did not correlate with any outcome, and this likely reflects its tight regulation. Although CDP–choline pathway substrates are cytosolic, its products are abundant in plasma and the elevations of its end lipid products including phosphatidylcholine, ceramide, sphingomyelin, and their diacylglycerol precursors were negatively correlated with gavage weight gain, placental efficiency, and fetal body and brain weights; the strength of these correlations varied with the metabolite and outcome. With respect to the PEMT pathway, its substrate phosphatidylethanolamine had similar negative correlations with placental efficiency and fetal body and brain weights. In contrast, most correlations for metabolites in the methyl donor pathway were positive. Dimethylglycine positively correlated with fetal body weight and gestational weight gain. Serine was positively correlated with placental weight, and the serine/glycine ratio was positively correlated with gestational and gavage weight gain, whereas glycine had a negative correlation with these.

To gain mechanistic insights into how choline might improve gestational outcomes, we also performed these correlations in the maternal liver, placenta, and fetal brain, given their respective contributions to choline status (Table 4 and Table 5 and Supplemental Figure S2). Most of the correlations were in the moderate range. For maternal liver, positive correlations were seen for the CDP–choline pathway substrate phosphocholine with fetal body weight and for CDP–choline with gestational weight gain and placental efficiency (Table 4). Hepatic CDP–choline was also negatively correlated with placental weight. In the PEMT pathway, CDP–ethanolamine was positively correlated with placental efficiency and fetal body and brain weights. Both SAM and the SAM/SAH ratio were positively correlated with gestational and gavage weight gain. Finally, with respect to other methyl donors, dimethylglycine was positively correlated with all outcomes except placental and fetal brain weight, and betaine was positively correlated with the fetal body weight. An elevated serine/glycine ratio was associated with higher placental weight and glycine with placental efficiency.

For the placenta, phosphocholine was negatively correlated with all outcomes except placental weight, and CDP–choline was negatively correlated with fetal body weight (Table 5 and Supplemental Figure S2). SAH was negatively correlated with fetal body and brain weights, whereas glycine was positively correlated with placental and fetal brain weights. Finally, fetal brain weight was positively correlated with ceramides, serine, and glycine but not with choline, SAM, SAH, or their ratios (Supplemental Table S5).

## 4. Discussion

**Figure 2:**
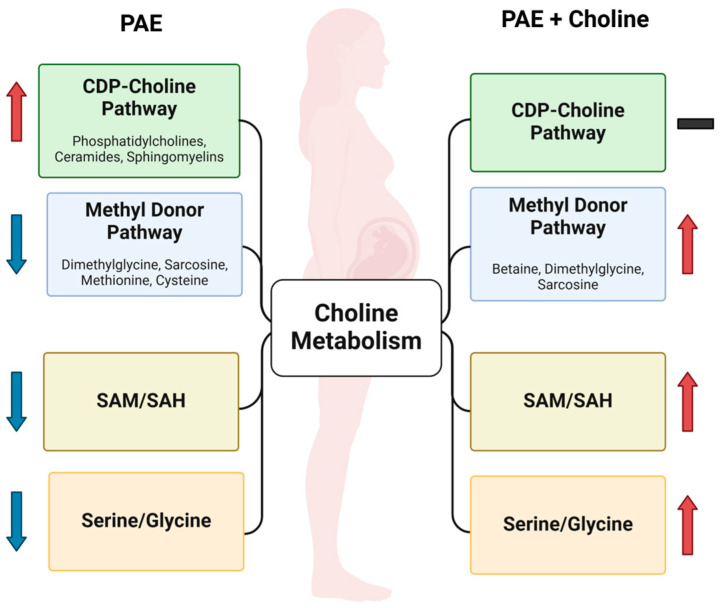
How alcohol affects choline metabolism across the maternal–fetal dyad and the role of supplemental choline. Alcohol increased CDP–choline pathway lipid products at the expense of methyl donor pools across the maternal–fetal dyad. This reduction in the methyl donor metabolites was partially normalized by choline. Created in BioRender. Petry, H. (https://www.biorender.com/, accessed on 25 February 2025).

These data present a snapshot of choline metabolism in the late-term maternal–fetal dyad, with a goal to generate novel insights into how alcohol interacts with choline status and the potential mechanism by which choline supplements mitigate alcohol’s effects. Using a model with homology to the most common diagnostic category, alcohol-related neurodevelopmental disorder (ARND), these data show that this alcohol exposure does not cause a choline deficiency per se, as the choline content in the maternal plasma and liver, placenta, and fetal brain is largely normal. Instead, we find that alcohol exposure redirects choline utilization into lipid biosynthesis at the expense of its critical role as a methyl donor, producing deficits in SAM and the ratios of SAM/SAH and serine/glycine. Choline supplementation addresses this redirection not by normalizing lipid synthesis, as these metabolites remain elevated, but rather by providing additional choline that repletes the insufficiency in methyl donors as seen in the choline-mediated increases in dimethylglycine, SAM/SAH, and serine/glycine ratios in maternal tissues, placenta, and the fetal brain (Figure 2). This metabolic shift is beneficial and is supported functionally by the negative correlations of choline-related lipid metabolites with gestational growth outcomes and the positive correlations of these growth outcomes with methylation-related metabolites. These data suggest that, as in the case of iron [48,49], PAE causes a functional insufficiency in methyl donor pools that is remediated by choline supplementation.

What might be mediating this diversion of choline away from methylation and toward lipid synthesis? The most likely candidate is the well-documented ability of chronic alcohol exposure to enhance hepatic lipid biosynthesis as well as to mobilize non-hepatic lipid stores for hepatic clearance [50,51]. Although our alcohol exposure is relatively moderate and was only from E 8.5 to E 17.5, we find that it is sufficient to nearly double the maternal hepatic triglyceride content (Saini, manuscript in preparation). The export of these hepatic lipids as lipoproteins (primarily very low-density lipoproteins (VLDLs)) requires a phosphatidylcholine coat, and the alcohol-associated steatosis is thought to reflect, in part, an inability to synthesize sufficient phosphatidylcholine to meet this heavy demand. Such an inadequacy in choline could limit the ability to meet the choline demands for phospholipid synthesis and could partly underlie the reductions in low-density lipoproteins reported for heavy drinkers who are pregnant [52] and a reduction in phospholipids in a rat model of PAE [53]. This alcohol-driven demand for the CDP–choline pathway would explain the ability of supplemental choline, betaine, and/or SAM to enhance hepatic VLDL release and attenuate the steatosis [54,55,56]. The rate-limiting step for choline entry into the CDP–choline pathway is choline kinase, which is upregulated under conditions that have a high demand for lipid biosynthesis such as embryogenesis and cancer [57]. Choline kinase is not subject to allosteric regulation and, thus, the flux of choline into phosphocholine, CDP–choline, and phosphatidylcholine is the most likely force drawing free choline into this pathway.

We speculate that this diversion of choline into lipid biosynthesis reduces the choline pools available for methylation purposes. This is supported by the alcohol-mediated reductions in methionine, SAM, SAM/SAH, serine, and serine/glycine and their improvement by supplemental choline. Methionine is the immediate source of methyl groups for SAM and is regenerated by choline-derived betaine. Methionine is also regenerated using folate and a serine-derived methyl group. Thus, under conditions when choline is limiting, as under the elevated lipid synthesis seen here, there is an increased demand to regenerate methionine and SAM via serine. Thus, a reduced serine/glycine ratio indicates an increased demand for this alternate methyl source, as glycine is produced by serine demethylation. Such reductions in serine, a non-essential amino acid, would be replenished in turn by generating serine from glucose at the level of the glycolytic intermediate 3-phosphoglycerate. We showed elsewhere [58] that in these same pregnancies, alcohol significantly reduces glucose levels in fasting maternal blood and liver, placenta, and fetal brain (range of 64–84% of CON). Such reductions would limit the glucose that is available to replenish serine and thereby further restrict methyl availability. Supplementation with choline would replenish the methyl pools and spare glucose and serine for anabolic purposes that support fetal growth, i.e., protein synthesis and the synthesis of purine and pyrimidines for DNA and RNA. Under this mechanism, it was expected that serine, serine/glycine, and SAM/SAH, as well as betaine and dimethylglycine, would positively correlate with fetal weight measures, and this is what we found. Lending clinical support is our recent metabolome analysis of alcohol-exposed human pregnancies, which found that plasma glycine negatively correlates with infant birth weight (R = −0.29) and head circumference (R = −0.36) [59]. It is also supported by the demonstration that a gestational supplement including folate, vitamin B12, and vitamin B6, which all participate in this alternate methyl pathway, conferred a cognitive benefit to the offspring of alcohol-exposed pregnancies that was not further enhanced by additional choline [10]. This emphasizes that micronutrients act in concert, and interventions involving choline might confer even greater benefits when accompanied by these B vitamins that further support methylation reactions.

Alcohol reduced the hepatic methyl pathway intermediates dimethylglycine and SAM/SAH, even though choline itself was unchanged, and this suggests that entry into this pathway was limiting. The mechanism underlying this is unknown. Choline entry into this pathway requires its mitochondrial oxidation to betaine by the choline and betaine aldehyde dehydrogenases. It is possible that competition between alcohol and choline oxidation might limit the availability of NAD reducing equivalents for betaine synthesis; all three of the relevant mitochondrial dehydrogenases have their highest activity in the liver. However, alcohol did not affect the hepatic abundance of betaine aldehyde and betaine, and hepatic lactate levels were actually reduced (81% of CON) [60], suggesting that this prenatal alcohol exposure does not appreciably alter the hepatic redox state. However, we cannot rule out that high-intensity or binge drinking could limit NAD availability to reduce betaine synthesis and SAM availability for methyl donation. Thus, the basis for the reductions in this pathway requires additional investigation.

These data also generate insights into which plasma biomarkers might be best suited to evaluate the methylation status in PAE. Although betaine and dimethylglycine are often used as biomarkers of choline status, and supplemental choline substantially raised their content above ALC and CON values, our data suggest that their abundance does not fully describe maternal choline needs given that methionine and the serine/glycine ratio were still below control values. This suggests that plasma methionine, serine, and the serine/glycine ratio may have better predictive value for assessing maternal–fetal choline status and for establishing the most effective choline dose to be used in clinical interventions for PAE. We further note that, although the liver is the primary site of choline utilization, the fasting plasma values for these amino acids mirrored their hepatic abundance and perhaps best reflect actual choline and methyl group needs.

This study has several limitations. This analysis was a single snapshot in the late term and may not reflect what is happening earlier in pregnancy. Many metabolites showed high variance, and others are tightly regulated, thereby only likely to show small changes in levels, which would only be found to be statistically significant with a very large sample size. As a result, this study may be underpowered to identify these between-group differences as statistically significant with an adjusted *p* value, and so we report effect sizes; this could be considered to be an exploratory hypothesis-generating study. The choline was administered by subcutaneous injection to assure consistent exposure; however, oral delivery is the physiologically relevant exposure and might confer a greater benefit as dietary choline is largely cleared by the liver. The choline dose used here was 175% of the rodent AI [39,61], and it did not fully normalize maternal–fetal methionine, serine, and serine/glycine [12,13], suggesting that this dose was insufficient and that a higher dose may be needed for full repletion. Because the metabolomic method used herein does not detect most metabolites in the folate pathway, the effects of alcohol and/or choline on this remain unclear.

A major unknown is the identity of the physiological and/or biochemical process(es) that are normalized by the supplemental choline in alcohol-exposed pregnancy. Methylation is a foundational metabolic process and methyl groups from folate and choline contribute to over 100 reactions including the synthesis of heme, amino acids, DNA and RNA nucleosides, carnitine for fat oxidation, polyamines to stabilize DNA and RNA structures, pseudouridylation for ribosomal RNAs, and histone and DNA modifications that regulate gene expression. It is highly unlikely that an alteration in any single one of these mediates alcohol’s adverse consequences. Rather, it may more closely resemble the euphemism of ‘death by a thousand cuts’, as many of these processes are altered in response to alcohol. This includes a repression of ribosome synthesis [62], fetal anemia [48,49], reductions in proliferation [63,64], mitochondrial activity [65], and a consistent hypomethylation at DNA regulatory sites [66].

## 5. Conclusions

In summary, we find that alcohol disrupts choline fate in a way that likely decreases methylation pools, creating an apparent choline insufficiency that may negatively affect fetal development. Although a 75% increase in daily choline may seem high, it was not sufficient to reverse all the effects of alcohol on choline fate. We suggest that future choline intervention studies include other micronutrients, including B vitamins, that also support methylation reactions.

## Figures and Tables

**Table 1:** Effect of alcohol on choline-related metabolites in maternal and fetal tissues (fold change (alcohol/control)).

Metabolites	Maternal Plasma	Maternal Liver	Placenta	Fetal Brain
Choline	0.9712	1.2533 (S)	1.0104	0.9710
CDP—Choline Pathway				
Phosphocholine	0.7948 (S)	1.2171 (S)	1.1312 (L)	1.0485
CDP—Choline	ND	1.0719	1.0790	1.2289 (L)
Phosphatidylcholines	1.1942 # (L)	1.0807	1.1382 *	1.0215
Ceramides	1.3921 *	1.4301 (L)	1.0957	0.9624
Sphingomyelins	1.2803 *	1.1231 (M)	1.1143 # (L)	1.0447
Diacylglycerols	1.0200	1.0426	1.0770	1.0810
PEMT Pathway				
CDP—Ethanolamine	ND	0.9994	1.2870 (L)	1.0831
Phosphatidylethanolamines	0.9738	1.2921 (L)	1.0941 # (L)	1.0174
SAM	ND	0.7289 # (L)	1.0374	1.0412
SAH	1.0902	0.9832	1.1854 (L)	0.9381
SAM/SAH Ratio	ND	0.7244 (L)	0.8476 (L)	1.1175 (M)
Methyl Donor Pathway				
Betaine	1.0415	1.0347	0.9805	1.0139
Dimethylglycine	0.9698	0.7121 # (L)	0.9282	0.7921 (L)
Sarcosine	ND	0.8742 (S)	ND	1.0518
Methionine	0.8574 (M)	0.8760 (S)	0.8720 (S)	0.8954 (S)
Cysteine	1.1189 (M)	0.9693	1.0701	1.3784 (L)
Other Metabolites				
Serine	0.7908 (M)	0.8944 (S)	0.7655 (L)	0.7988 # (L)
Glycine	0.9550	1.1310 (S)	0.9433	0.9305
Serine/Glycine Ratio	0.7733 (M)	0.8288 (M)	0.8132 # (L)	0.8578 # (L)
TMAO	0.4071 (L)	0.3612 (L)	0.5548 (L)	0.7854 (L)

* FDR < 0.05, # 0.05 < FDR < 0.1. CDP—cytidine diphosphate; FDR—False Discovery Rate; (L)—large effect size; (M)—medium effect size; (S)—small effect size; ND—not detected; PEMT—Phosphatidylethanolamine N-Methyltransferase; SAH—S-Adenosyl Homocysteine; SAM—S-Adenosyl Methionine; TMAO—trimethylamine N-oxide.

**Table 2:** Effect of choline on choline-related metabolites in alcohol-exposed maternal–fetal tissues (fold change (alcohol + choline/alcohol)).

Metabolites	Maternal Plasma	Maternal Liver	Placenta	Fetal Brain
Choline	1.0248	1.1008 (S)	1.0250	0.9404
CDP—Choline Pathway				
Phosphocholine	0.7661 (M)	1.4036 (L)	0.9298	0.9274
CDP–Choline	ND	1.2820 (M)	0.8747 (M)	0.6625 (L)
Phosphatidylcholines	0.9740	0.9649	0.9383	1.0264
Ceramides	1.0108	1.0024	0.9158	1.0672
Sphingomyelins	0.9367	0.8985 (S)	0.9484	1.0772
Diacylglycerols	0.9670	1.1038 (S)	0.9659	1.1070 (M)
PEMT Pathway				
CDP–Ethanolamine	ND	0.9225	0.7928 (L)	0.8813 # (L)
Phosphatidylethanolamines	1.1206 (M)	0.9004	1.0380	1.0476
SAM	ND	1.2648 (L)	0.9806	0.9132
SAH	0.6389 (L)	1.1217 (M)	0.9513	0.9678
SAM/SAH Ratio	ND	1.1492 (M)	1.0375	0.9411
Methyl Donor Pathway				
Betaine	1.0372	1.5433 (L)	0.9111	1.0471
Dimethylglycine	1.3635 (L)	1.6997 (L)	1.4798 (L)	1.4138 (L)
Sarcosine	ND	1.7170 (M)	ND	1.5091 (L)
Methionine	0.9929	1.0273	1.0653	0.8438 (L)
Cysteine	0.9578	1.0756	1.1522 (M)	0.8161 (L)
Other Metabolites				
Serine	1.8111 (M)	1.0910 (M)	1.3459 (L)	1.0598
Glycine	0.8497 (S)	0.9271	1.0395	1.0404
Serine/Glycine Ratio	1.3899 (L)	1.1313 (M)	1.2922 *	1.0237
TMAO	2.2548 (L)	2.4409 (L)	1.6200 (L)	1.2773 (L)

* FDR < 0.05, # 0.05 < FDR < 0.1. CDP—cytidine diphosphate; FDR—False Discovery Rate; (L)—large effect size; (M)—medium effect size; (S)—small effect size; ND—not detected; PEMT—Phosphatidylethanolamine N-Methyltransferase; SAH—S-Adenosyl Homocysteine; SAM—S-Adenosyl Methionine; TMAO—trimethylamine N-oxide.

**Table 3:** Spearman correlation analysis comparing maternal plasma metabolites with pregnancy and fetal outcomes.

Metabolites	Gestational Weight Gain	Gavage Weight Gain	Placenta Weight	Placental Efficiency	Fetal Body Weight	Fetal Brain Weight
Choline	0.1778	0.1803	−0.0897	0.0990	0.0512	0.0140
CDP—Choline Pathway						
Phosphocholine	0.2773	0.2424	0.2178	−0.0241	0.1136	0.3218 #
Phosphatidylcholines	−0.2798	−0.3917 *	0.1786	−0.2690	−0.1641	−0.2412
Ceramides	−0.0783	−0.1577	−0.0463	−0.1094	−0.1377	−0.3158 #
Sphingomyelins	−0.2650	−0.4126 *	0.2540	−0.3768 *	−0.1968	−0.2446
Diacylglycerols	−0.0818	−0.0355	−0.0897	−0.4212 *	−0.6905 *	−0.6302 *
PEMT Pathway						
Phosphatidylethanolamines	0.0926	−0.0411	0.0636	−0.3384 #	−0.4353 *	−0.5893 *
SAH	0.1382	0.2368	−0.0410	0.0493	−0.1050	0.0995
Methyl Donor Pathway						
Betaine	0.1714	0.1106	0.0599	0.0645	0.1345	0.1237
Dimethylglycine	0.3734 *	0.2902	−0.0577	0.3084	0.4062 *	0.1185
Methionine	−0.1310	−0.2074	0.4166 *	−0.4251 *	−0.2749	−0.1798
Cysteine	0.1635	0.1833	0.0480	−0.0833	−0.1919	−0.916
Other Metabolites						
Serine	0.2197	0.1084	0.3124 #	−0.2621	−0.1439	−0.919
Glycine	−0.3261 #	−0.3774 *	0.3067	−0.1360	0.0660	0.0234
Serine/Glycine Ratio	0.4414 *	0.3764 *	−0.0567	0.0419	−0.0446	−0.0751

Spearman correlation coefficient r_s_; * *p* < 0.05, 0.05 < # < 0.1; CDP—cytidine diphosphate; PEMT—Phosphatidylethanolamine N-Methyltransferase; SAH—S-Adenosyl Homocysteine.

**Table 4:** Spearman correlation analysis of maternal liver metabolites with pregnancy and fetal outcomes.

Metabolites	Gestational Weight Gain	Gavage Weight Gain	Placenta Weight	Placental Efficiency	Fetal Body Weight	Fetal Brain Weight
Choline	−0.1290	−0.2788	0.3618 *	0.0070	0.2955	−0.0073
CDP–Choline Pathway						
Phosphocholine	0.3065 #	0.1397	−0.0290	0.2804	0.3259 #	−0.0693
CDP–Choline	0.4428 *	0.4788 *	−0.4589 *	0.5161 *	0.2064	0.0268
Phosphatidylcholines	−0.2016	−0.1477	0.1133	−0.2621	−0.2544	−0.0524
Ceramides	−0.1382	−0.1111	0.0658	−0.2013	−0.1776	−0.0867
Sphingomyelins	−0.1488	−0.1362	0.2383	−0.3691 *	−0.2738	0.0000
Diacylglycerols	0.0836	0.1487	0.0359	−0.1191	−0.2034	−0.0777
PEMT Pathway						
CDP–Ethanolamine	0.1716	0.1281	−0.1188	0.3684 *	0.3886 *	0.3185 #
Phosphatidylethanolamines	−0.1899	−0.2040	0.2353	−0.2874	−0.1811	−0.0183
SAM	0.3149 #	0.3452 #	−0.1129	0.1551	0.0070	0.0957
SAH	−0.0620	0.0159	−0.0139	0.1136	−0.0839	−0.2551
SAM/SAH Ratio	0.4175 *	0.3957 *	−0.0850	0.1367	0.1342	0.2896
Methyl Donor Pathway						
Betaine	0.1004	0.0335	0.0872	0.2720	0.3823 *	0.2199
Dimethylglycine	0.3592 *	0.3199 #	0.0022	0.3156 #	0.3427 #	0.0935
Sarcosine	0.1415	0.1523	−0.0334	0.0400	−0.0990	−0.2093
Methionine	−0.0850	−0.1199	0.2093	−0.1268	0.0396	0.0773
Cysteine	0.0920	0.0321	0.1789	−0.1932	−0.0308	−0.0323
Other Metabolites						
Serine	0.0565	0.0524	0.2196	−0.0297	0.1320	0.1485
Glycine	−0.0583	0.0053	−0.1987	0.3068 #	0.2012	0.0689
Serine/Glycine Ratio	0.1338	0.0847	0.3284 #	−0.2137	0.0363	0.1657

Spearman correlation coefficient r_s_; * *p* < 0.05, 0.05 < # < 0.1; CDP—cytidine diphosphate; PEMT—Phosphatidylethanolamine N-Methyltransferase; SAH—S-Adenosyl Homocysteine; SAM—S-Adenosyl Methionine.

**Table 5:** Spearman correlation analysis comparing placental and fetal brain metabolites with pregnancy outcomes.

	Placenta Metabolites				Fetal Brain Metabolites
Metabolites	Placenta Weight	Placental Efficiency	Fetal Body Weight	Fetal Brain Weight	Fetal Brain Weight
Choline	−0.0106	0.1092	0.0766	−0.1338	0.1598
CDP–Choline Pathway					
Phosphocholine	−0.1268	−0.2478	−0.4930 *	−0.4267 *	−0.2786
CDP–Choline	−0.2218	−0.1125	−0.3152 #	0.0319	0.0649
Phosphatidylcholines	−0.2033	0.1641	−0.0422	−0.1030	−0.1276
Ceramides	−0.0216	0.0099	−0.0018	0.1639	0.3079 *
Sphingomyelins	−0.1345	0.2054	0.0338	−0.0637	−0.1430
Diacylglycerols	−0.1331	0.0696	−0.0663	−0.0554	0.2731
PEMT Pathway					
CDP–Ethanolamine	−0.1298	−0.1617	−0.2746	−0.0726	0.1697
Phosphatidylethanolamines	−0.0488	0.1712	0.1790	−0.0290	−0.1107
SAM	0.0323	−0.3633 *	−0.2859	−0.1972	0.1378
SAH	−0.2570	−0.0572	−0.3189 #	−0.3350 #	−0.0473
SAM/SAH Ratio	0.2064	−0.2603	−0.0418	0.0051	0.1609
Methyl Donor Pathway					
Betaine	0.0616	0.1870	0.2067	0.3996 *	−0.2588
Dimethylglycine	0.1492	0.2672	0.4443 *	0.1675	0.1378
Sarcosine	ND	ND	ND	ND	−0.3226 #
Methionine	0.4025 *	−0.2885	−0.0594	0.0392	−0.0209
Cysteine	−0.1221	0.2166	0.1924	0.0740	−0.0920
Other Metabolites					
Serine	0.1767	0.0040	0.1573	0.1514	0.3248 #
Glycine	0.3204 #	−0.1010	0.1338	0.3728 *	0.4010 *
Serine/Glycine Ratio	0.0297	0.0942	0.1782	0.0048	−0.0817

Spearman correlation coefficient r_s_; * *p* < 0.05, 0.05 < # < 0.1; CDP—cytidine diphosphate; ND- not detected; PEMT—Phosphatidylethanolamine N-Methyltransferase; SAH—S-Adenosyl Homocysteine; SAM—S-Adenosyl Methionine.

## Data Availability

The original contributions presented in this study are included in the article/Supplementary Materials. Further inquiries can be directed to the corresponding authors.

## Supplementary Materials

The following supporting information can be downloaded at: https://www.mdpi.com/article/10.3390/metabo15050289/s1, Figure S1: Change in 5-methyltetrahydrofolate (5MeTHF); Figure S2: Choline-Related Metabolites Correlated with Pregnancy Outcomes; Figure S3: Maternal Plasma Choline-Related Metabolites Abundance Relative to Control; Figure S4: Maternal Liver Choline-Related Metabolites Abundance Relative to Control; Figure S5: Placenta Choline-Related Metabolites Abundance Relative to Control.; Figure S6: Fetal Brain Choline-Related Metabolites Abundance Relative to Control; Table S1: Fold-Change of All Choline-Related Metabolites; Table S2: Choline-Related Lipid Metabolites and Amount Changed By at Least 10%; Table S3: Fold-Change (Alcohol + Choline/Control) in Choline-Related Metabolites in Maternal-Fetal Tissues; Table S4: Fold-Change (Control + Choline/Control) in Choline-Related Metabolites in Maternal-Fetal Tissues; Table S5: Gestational and Fetal Weight Outcomes

## Abbreviations

The following abbreviations are used in this manuscript:

5MeTHF	5-Methyltetrahydrofolate
AI	Adequate intake
ALC	Alcohol-exposed
ARND	Alcohol-related neurodevelopment disorder
AUC	Ares under the curve
CDH	Choline dehydrogenase
CDP	Cytidine diphosphate
Cho	Choline-treated
CK	Choline kinase
CON	Control
DNA	Deoxyribonucleic acid
E	Embryonic day
FASD	Fetal Alcohol Spectrum Disorder
FC	Fold change
FDR	False discovery rate
g	Gram
kg	Kilogram
mg	Milligram
NAD	Nicotinamide adenine dinucleotide
ND	Not detected
PAE	Prenatal alcohol exposure
PEMT	Phosphatidylethanolamine methyltransferase
RNA	Ribonucleic acid
SAH	S-adenosylhomocysteine
SAM	S-adenosylmethionine
THF	Tetrahydrofolate
TMAO	Trimethylamine N-oxide
UPLC-MS	Ultra-high-performance liquid chromatography-mass spectrometry
VLDL	Very low-density lipoprotein
