# Supplementary material for: Alcohol Exposure May Increase Prenatal Choline Needs Through Redirection of Choline into Lipid Synthesis Rather than Methyl Donation
Source: Metabolites. 2025 Apr 24;15(5):289. doi: 10.3390/metabo15050289 (PMC12113322; doi:10.3390/metabo15050289)
Supplement: Supplementary file 1 [file metabolites-15-00289-s001.zip › Supplemental Table 1.pdf]

Table S1. Fold-Change of All Choline-Related Metabolites

Metbaolite	Sub Pathway	Fold Change (ALC/CON)	p-value	FDR	Fold Change (ALC + CHO/ALC)	p-value	FDR	Fold Change (CON + CHO/CON)	p-value	FDR	Fold Change (ALC + CHO/CON)	p-value	FDR
**MATERNAL PLASMA**													
Choline	Phospholipid Metabolism	0.9712	0.7984	0.9075	1.0248	0.7789	0.9321	1.0931	0.2824	0.9075	0.9953	0.9551	0.9867
CDP-Choline Pathway													
Phosphocholine	Phospholipid Metabolism	0.7948	0.3823	0.6305	0.7661	0.1893	0.6773	1.0124	0.3450	0.9075	0.6089	0.0289	0.1506
1,2-dilinoleoyl-GPC (18:2/18:2)	Phosphatidylcholine	0.6309	0.2786	0.5365	0.8857	0.5358	0.8430	0.6610	0.2824	0.9075	0.5588	0.1520	0.3931
1,2-dipalmitoyl-GPC (16:0/16:0)	Phosphatidylcholine	0.9400	0.3823	0.6305	0.8849	0.1893	0.6773	0.9957	0.9497	0.9964	0.8318	0.0401	0.1948
1-linoleoyl-2-arachidonoyl-GPC (18:2/20:4n6)*	Phosphatidylcholine	1.4878	0.0281	0.1434	0.9125	0.6943	0.9003	1.1604	1.0000	1.0000	1.3575	0.0541	0.2235
1-linoleoyl-2-linolenoyl-GPC (18:2/18:3)*	Phosphatidylcholine	0.8550	0.7209	0.8743	0.7757	0.1893	0.6773	0.7126	0.4908	0.9402	0.6632	0.4634	0.7259
1-myristoyl-2-arachidonoyl-GPC (14:0/20:4)*	Phosphatidylcholine	3.0882	0.0002	0.0225	0.7790	0.1893	0.6773	1.1717	0.2824	0.9075	2.4056	0.0006	0.0223
1-myristoyl-2-palmitoyl-GPC (14:0/16:0)	Phosphatidylcholine	1.5490	0.0104	0.0908	0.7189	0.0205	0.3186	0.8127	0.5728	0.9537	1.1135	0.4634	0.7259
1-oleoyl-2-docosahexaenoyl-GPC (18:1/22:6)*	Phosphatidylcholine	1.2230	0.0830	0.2670	1.0321	0.7789	0.9321	1.0792	0.5728	0.9537	1.2623	0.0401	0.1948
1-oleoyl-2-linoleoyl-GPC (18:1/18:2)*	Phosphatidylcholine	0.6797	0.8785	0.9479	1.0515	0.7789	0.9321	0.6673	0.5728	0.9537	0.7147	0.7789	0.9091
1-palmitoyl-2-arachidonoyl-GPC (16:0/20:4n6)	Phosphatidylcholine	1.3081	0.0070	0.0713	0.9932	0.7789	0.9321	1.2212	0.0200	0.6907	1.2992	0.0022	0.0372
1-palmitoyl-2-dihomo-linolenoyl-GPC (16:0/20:3n3 or 6)*	Phosphatidylcholine	0.8773	0.6355	0.8270	1.1513	0.6943	0.9003	1.0947	0.6499	0.9555	0.9628	0.9537	0.9867
1-palmitoyl-2-docosahexaenoyl-GPC (16:0/22:6)	Phosphatidylcholine	1.0717	0.3823	0.6305	1.0802	0.6126	0.8820	1.2003	0.1419	0.8655	1.1576	0.0721	0.2579
1-palmitoyl-2-gamma-linolenoyl-GPC (16:0/18:3n6)*	Phosphatidylcholine	1.5792	0.0019	0.0409	0.9482	0.7789	0.9321	1.1758	0.1419	0.8655	1.4974	0.0037	0.0497
1-palmitoyl-2-linoleoyl-GPC (16:0/18:2)	Phosphatidylcholine	0.9974	0.9591	0.9780	1.0211	0.7789	0.9321	1.0874	1.0000	1.0000	1.0184	0.9551	0.9867
1-palmitoyl-2-oleoyl-GPC (16:0/18:1)	Phosphatidylcholine	1.2590	0.0207	0.1188	0.9987	0.5358	0.8430	1.2196	0.2284	0.9075	1.2573	0.0205	0.1317
1-palmitoyl-2-palmitoleoyl-GPC (16:0/16:1)*	Phosphatidylcholine	1.7004	0.0030	0.0486	0.8565	0.1206	0.6030	1.0882	0.3450	0.9075	1.4563	0.0289	0.1506
1-palmitoyl-2-stearoyl-GPC (16:0/18:0)	Phosphatidylcholine	1.1659	0.1304	0.3535	0.8384	0.0939	0.6030	1.0905	0.7546	0.9964	0.9776	0.7789	0.9091
1-stearoyl-2-arachidonoyl-GPC (18:0/20:4)	Phosphatidylcholine	1.5263	0.0019	0.0409	0.9054	0.1893	0.6773	1.2258	0.1812	0.9070	1.3819	0.0012	0.0279
1-stearoyl-2-docosahexaenoyl-GPC (18:0/22:6)	Phosphatidylcholine	1.3761	0.0019	0.0409	0.9208	0.2319	0.7264	1.1956	0.6620	0.9555	1.2671	0.0059	0.0674
1-stearoyl-2-linoleoyl-GPC (18:0/18:2)*	Phosphatidylcholine	1.1735	0.0650	0.2363	0.9285	0.3969	0.7788	1.0822	0.9497	0.9964	1.0895	0.3969	0.6636
1-stearoyl-2-oleoyl-GPC (18:0/18:1)	Phosphatidylcholine	1.7201	0.0030	0.0486	0.9249	0.5358	0.8430	1.3428	0.5728	0.9537	1.5909	0.0022	0.0372
ceramide (d18:2/24:1, d18:1/24:2)*	Ceramides	1.8776	0.0019	0.0409	1.0209	0.8665	0.9628	1.4551	0.2284	0.9075	1.9168	0.0022	0.0372
N-palmitoyl-sphingosine (d18:1/16:0)	Ceramides	1.3930	0.0207	0.1188	0.9701	0.6943	0.9003	1.2360	0.4136	0.9296	1.3513	0.0541	0.2235
N-stearoyl-sphingosine (d18:1/18:0)*	Ceramides	1.3940	0.0830	0.2670	1.1174	0.2810	0.7458	1.5029	0.0813	0.7661	1.5575	0.0140	0.1037
N-palmitoyl-sphinganine (d18:0/16:0)	Ceramides	0.8407	0.6454	0.8270	0.9356	1.0000	1.0000	0.7420	0.4136	0.9296	0.7866	0.5358	0.7752
behenoyl sphingomyelin (d18:1/22:0)*	Sphingomyelins	1.3589	0.0148	0.0999	0.9132	0.4634	0.8093	1.3401	0.1419	0.8655	1.2409	0.0541	0.2235
hydroxypalmitoyl sphingomyelin (d18:1/16:0(OH))	Sphingomyelins	1.0262	0.3282	0.5796	0.9369	0.1893	0.6773	1.0122	0.7546	0.9964	0.9615	0.8665	0.9512
lignoceroyl sphingomyelin (d18:1/24:0)	Sphingomyelins	1.3324	0.0207	0.1188	0.8902	0.2810	0.7458	1.3223	0.4136	0.9296	1.1860	0.1893	0.4447
palmitoyl sphingomyelin (d18:1/16:0)	Sphingomyelins	1.1788	0.0830	0.2670	0.9945	0.6943	0.9003	1.2722	0.1812	0.9070	1.1723	0.3969	0.6636
sphingomyelin (d17:1/16:0, d18:1/15:0, d16:1/17:0)*	Sphingomyelins	1.1295	0.4418	0.6720	0.9774	0.8665	0.9628	1.1013	0.9497	0.9964	1.1040	0.5358	0.7752
sphingomyelin (d18:1/14:0, d16:1/16:0)*	Sphingomyelins	1.3226	0.0148	0.0999	0.9847	0.6943	0.9003	1.2794	0.7546	0.9964	1.3023	0.0939	0.3026
sphingomyelin (d18:1/17:0, d17:1/18:0, d19:1/16:0)	Sphingomyelins	1.2489	0.1605	0.3900	0.9252	0.6943	0.9003	1.3784	0.2284	0.9075	1.1555	0.2319	0.4918
sphingomyelin (d18:1/18:1, d18:2/18:0)	Sphingomyelins	1.1359	0.3823	0.6305	0.9012	0.3969	0.7788	1.1826	0.4908	0.9402	1.0237	0.7789	0.9091
sphingomyelin (d18:1/20:0, d16:1/22:0)*	Sphingomyelins	1.4147	0.0070	0.0713	0.8887	0.4634	0.8093	1.4113	0.1419	0.8655	1.2572	0.0721	0.2579
sphingomyelin (d18:1/20:1, d18:2/20:0)*	Sphingomyelins	1.0694	0.7209	0.8743	0.9080	0.5358	0.8430	1.1483	0.4908	0.9402	0.9710	0.5358	0.7752
sphingomyelin (d18:1/21:0, d17:1/22:0, d16:1/23:0)*	Sphingomyelins	1.4501	0.0207	0.1188	0.9086	0.3357	0.7681	1.3302	0.2284	0.9075	1.3175	0.0939	0.3026
sphingomyelin (d18:1/22:1, d18:2/22:0, d16:1/24:1)*	Sphingomyelins	1.3170	0.0104	0.0908	0.9071	0.2810	0.7458	1.2414	0.7546	0.9964	1.1946	0.3357	0.6236
sphingomyelin (d18:1/24:1, d18:2/24:0)*	Sphingomyelins	1.4195	0.0070	0.0713	0.8964	0.3969	0.7788	1.4217	0.7546	0.9964	1.2724	0.1893	0.4447
sphingomyelin (d18:2/16:0, d18:1/16:1)*	Sphingomyelins	1.2695	0.0207	0.1188	0.9648	0.6126	0.8820	1.2456	0.9497	0.9964	1.2248	0.1893	0.4447
sphingomyelin (d18:2/18:1)*	Sphingomyelins	1.0844	0.8785	0.9479	0.9595	0.8665	0.9628	1.2526	0.9497	0.9964	1.0406	0.9551	0.9867
sphingomyelin (d18:2/23:0, d18:1/23:1, d17:1/24:1)*	Sphingomyelins	1.3586	0.0104	0.0908	0.9372	0.6126	0.8820	1.2515	0.8518	0.9964	1.2733	0.1206	0.3496
sphingomyelin (d18:2/23:1)*	Sphingomyelins	1.1733	0.3282	0.5796	0.9760	0.8665	0.9628	1.1688	0.8518	0.9964	1.1452	0.6126	0.8141
sphingomyelin (d18:2/24:1, d18:1/24:2)*	Sphingomyelins	1.2579	0.0650	0.2363	0.9468	0.6126	0.8820	1.2956	0.6620	0.9555	1.1910	0.2319	0.4918
sphingomyelin (d18:2/24:2)*	Sphingomyelins	1.0947	0.3282	0.5796	1.0262	0.9551	0.9862	1.1549	0.4908	0.9402	1.1234	0.2319	0.4918
stearoyl sphingomyelin (d18:1/18:0)	Sphingomyelins	1.2038	0.2786	0.5365	0.8824	0.3357	0.7681	1.3395	0.1079	0.8245	1.0622	0.7789	0.9091
tricosanoyl sphingomyelin (d18:1/23:0)*	Sphingomyelins	1.6774	0.0207	0.1188	0.8520	0.3357	0.7681	1.7193	0.3450	0.9075	1.4292	0.0205	0.1317
palmitoyl dihydrosphingomyelin (d18:0/16:0)*	Sphingomyelins	1.0107	0.6454	0.8270	0.9914	0.4634	0.8093	1.2841	0.6620	0.9555	1.0020	0.8665	0.9512
sphingomyelin (d18:0/18:0, d19:0/17:0)*	Sphingomyelins	1.1980	0.2345	0.4837	0.9159	0.5358	0.8430	1.8768	0.0293	0.7661	1.0973	0.8665	0.9512
diacylglycerol (16:1/18:2 [2], 16:0/18:3 [1])*	Diacylglycerols	1.5137	0.1267	0.3535	0.7580	0.1828	0.6773	0.9501	0.9483	0.9964	1.1129	0.9070	0.9867
linoleoyl-arachidonoyl-glycerol (18:2/20:4) [1]*	Diacylglycerols	1.1352	0.3282	0.5796	1.2540	0.1893	0.6773	1.2782	0.1079	0.8245	1.4235	0.0721	0.2579
linoleoyl-arachidonoyl-glycerol (18:2/20:4) [2]*	Diacylglycerols	0.9273	1.0000	1.0000	1.2416	0.5358	0.8430	1.2427	0.2824	0.9075	1.1514	0.6126	0.8141
linoleoyl-docosahexaenoyl-glycerol (18:2/22:6) [1]*	Diacylglycerols	1.2866	0.1605	0.3900	1.3001	0.2810	0.7458	1.3214	0.4136	0.9296	1.6727	0.0541	0.2235
linoleoyl-linolenoyl-glycerol (18:2/18:3) [1]*	Diacylglycerols	0.6511	0.4418	0.6720	0.6470	0.2319	0.7264	0.5258	0.3450	0.9075	0.4213	0.1520	0.3931
linoleoyl-linolenoyl-glycerol (18:2/18:3) [2]*	Diacylglycerols	0.6719	0.4418	0.6720	0.6543	0.9551	0.9862	0.5587	0.3450	0.9075	0.4396	0.2319	0.4918
linoleoyl-linoleoyl-glycerol (18:2/18:2) [1]*	Diacylglycerols	0.7757	0.6454	0.8270	0.7739	0.3969	0.7788	0.7770	0.4908	0.9402	0.6004	0.3357	0.6236
oleoyl-arachidonoyl-glycerol (18:1/20:4) [1]*	Diacylglycerols	1.3521	0.0650	0.2363	1.0852	0.6943	0.9003	1.2071	0.3450	0.9075	1.4673	0.0541	0.2235
oleoyl-arachidonoyl-glycerol (18:1/20:4) [2]*	Diacylglycerols	1.4297	0.0499	0.2001	1.0386	0.9551	0.9862	1.2264	0.4136	0.9296	1.4849	0.0721	0.2579
oleoyl-linoleoyl-glycerol (18:1/18:2) [1]	Diacylglycerols	1.1142	0.7984	0.9075	0.8247	0.0939	0.6030	1.0667	0.9497	0.9964	0.9189	0.8665	0.9512
oleoyl-linoleoyl-glycerol (18:1/18:2) [2]	Diacylglycerols	1.1440	0.5054	0.7407	0.7963	0.1206	0.6030	1.0888	0.9497	0.9964	0.9110	0.8665	0.9512
palmitoleoyl-arachidonoyl-glycerol (16:1/20:4) [2]*	Diacylglycerols	1.6994	0.1501	0.3900	1.2860	0.9538	0.9862	1.6846	0.0312	0.7661	1.9800	0.0167	0.1211
palmitoleoyl-linoleoyl-glycerol (16:1/18:2) [1]*	Diacylglycerols	2.1448	0.0036	0.0581	0.8300	0.5358	0.8430	1.8493	0.2400	0.9075	1.7802	0.0228	0.1449
palmitoyl-arachidonoyl-glycerol (16:0/20:4) [1]*	Diacylglycerols	0.8738	0.5283	0.7727	1.3118	0.0930	0.6030	1.0297	0.9497	0.9964	1.1938	0.6126	0.8141
palmitoyl-arachidonoyl-glycerol (16:0/20:4) [2]*	Diacylglycerols	1.0629	0.4295	0.6720	0.8932	0.9538	0.9862	0.9709	0.9483	0.9964	1.0191	0.7703	0.9091
palmitoyl-docosahexaenoyl-glycerol (16:0/22:6) [1]*	Diacylglycerols	1.5563	0.0238	0.1349	0.9742	0.9078	0.9862	0.9715	0.7940	0.9964	1.4984	0.1466	0.3931
palmitoyl-linoleoyl-glycerol (16:0/18:2) [1]*	Diacylglycerols	0.9560	0.9591	0.9780	0.8568	0.4634	0.8093	0.9878	0.9497	0.9964	0.8191	0.5358	0.7752
palmitoyl-linoleoyl-glycerol (16:0/18:2) [2]*	Diacylglycerols	1.0325	0.9591	0.9780	0.7919	0.2319	0.7264	1.0289	0.9497	0.9964	0.8176	0.4634	0.7259
stearoyl-arachidonoyl-glycerol (18:0/20:4) [1]*	Diacylglycerols	0.9456	0.7984	0.9075	1.1413	0.5358	0.8430	1.0789	0.6620	0.9555	1.0792	0.6943	0.8758
stearoyl-arachidonoyl-glycerol (18:0/20:4) [2]*	Diacylglycerols	0.9606	0.7923	0.9075	1.0430	0.6852	0.9003	1.1926	0.7960	0.9964	0.9926	1.0000	1.0000
PEMT Pathway													
1,2-dilinoleoyl-GPE (18:2/18:2)*	Phosphatidylethanolamines	0.5228	0.0650	0.2363	0.9228	0.7789	0.9321	0.6021	0.0813	0.7661	0.4824	0.0541	0.2235
1-linoleoyl-2-arachidonoyl-GPE (18:2/20:4)*	Phosphatidylethanolamines	1.1072	0.7209	0.8743	0.7017	0.0037	0.1815	0.8501	0.4908	0.9402	0.7769	0.3357	0.6236
1-oleoyl-2-arachidonoyl-GPE (18:1/20:4)*	Phosphatidylethanolamines	1.2242	0.1304	0.3535	0.9868	0.8665	0.9628	1.0105	1.0000	1.0000	1.2081	0.0721	0.2579
1-oleoyl-2-docosahexaenoyl-GPE (18:1/22:6)*	Phosphatidylethanolamines	1.2907	0.1304	0.3535	1.0883	0.2319	0.7264	1.0793	0.9497	0.9964	1.4047	0.0140	0.1037

1-oleoyl-2-linoleoyl-GPE (18:1/18:2)*	Phosphatidylethanolamines	0.4996	0.0379	0.1737	1.0492	0.8665	0.9628	0.6449	0.0813	0.7661	0.5242	0.0289	0.1506
1-palmitoyl-2-arachidonoyl-GPE (16:0/20:4)*	Phosphatidylethanolamines	1.0467	0.7209	0.8743	1.2266	0.3357	0.7681	1.1433	0.2824	0.9075	1.2839	0.0541	0.2235
1-palmitoyl-2-docosahexaenoyl-GPE (16:0/22:6)*	Phosphatidylethanolamines	0.9703	0.8785	0.9479	1.3336	0.0289	0.3768	1.2190	0.1812	0.9070	1.2939	0.0401	0.1948
1-palmitoyl-2-linoleoyl-GPE (16:0/18:2)	Phosphatidylethanolamines	0.5528	0.0148	0.0999	1.0734	0.6943	0.9003	0.7576	0.2824	0.9075	0.5934	0.0289	0.1506
1-palmitoyl-2-oleoyl-GPE (16:0/18:1)	Phosphatidylethanolamines	0.8463	0.4418	0.6720	0.8125	0.2319	0.7264	0.8074	0.3450	0.9075	0.6877	0.1206	0.3496
1-stearoyl-2-arachidonoyl-GPE (18:0/20:4)	Phosphatidylethanolamines	1.2684	0.0499	0.2001	1.0612	0.7789	0.9321	1.1657	0.4908	0.9402	1.3460	0.0289	0.1506
1-stearoyl-2-docosahexaenoyl-GPE (18:0/22:6)*	Phosphatidylethanolamines	1.0890	0.5737	0.7942	1.1954	0.1206	0.6030	1.1022	0.4136	0.9296	1.3018	0.0939	0.3026
1-stearoyl-2-linoleoyl-GPE (18:0/18:2)*	Phosphatidylethanolamines	0.6690	0.1049	0.3100	0.9636	0.7789	0.9321	0.8067	0.3450	0.9075	0.6447	0.0401	0.1948
1-stearoyl-2-oleoyl-GPE (18:0/18:1)	Phosphatidylethanolamines	0.6937	0.1949	0.4315	0.9635	0.7789	0.9321	0.8499	0.6620	0.9555	0.6684	0.1520	0.3931
Methyl Donor Pathway													
Betaine	Glycine, Serine and Threonine Metabolism	1.0415	0.7209	0.8743	1.0372	0.6943	0.9003	1.2742	0.0813	0.7661	1.0802	0.5358	0.7752
Dimethylglycine	Glycine, Serine and Threonine Metabolism	0.9698	0.7984	0.9075	1.3635	0.0059	0.2155	1.2576	0.0593	0.7661	1.3223	0.0093	0.0828
Sarcosine	Glycine, Serine and Threonine Metabolism				0.6779	0.1407	0.6526						
Methionine	Methionine, Cysteine, SAM and Taurine Metabolism	0.8574	0.3282	0.5796	0.9929	1.0000	1.0000	0.8410	0.2284	0.9075	0.8513	0.3969	0.6636
Cysteine	Methionine, Cysteine, SAM and Taurine Metabolism	1.1189	0.2345	0.4837	0.9758	0.2810	0.7458	1.3709	0.0813	0.7661	1.0918	0.8665	0.9512
Other Metabolites													
S-adenosylhomocysteine (SAH)	Methionine, Cysteine, SAM and Taurine Metabolism	1.0902	0.3823	0.6305	0.6389	0.0128	0.2917	1.6805	0.4136	0.9296	0.6965	0.0239	0.1481
Serine	Glycine, Serine and Threonine Metabolism	0.7908	0.1605	0.3900	1.1811	0.2319	0.7264	1.0585	0.8518	0.9964	0.9340	0.3969	0.6636
Glycine	Glycine, Serine and Threonine Metabolism	0.9550	0.9591	0.9780	0.8497	0.4634	0.8093	0.8486	0.6620	0.9555	0.8115	0.4634	0.7259
trimethylamine N-oxide (TMAO)	Phospholipid Metabolism	0.2779	0.0207	0.1188	2.2548	0.0037	0.1815	0.6350	0.5728	0.9537	0.6267	0.4634	0.7259
**MATERNAL LIVER**													
Choline	Phospholipid Metabolism	1.2533	0.3823	0.6490	1.1008	0.4418	0.8963	1.0937	0.0830	0.7889	1.3796	0.0019	0.0480
CDP-Choline Pathway													
Phosphocholine	Phospholipid Metabolism	1.2171	0.5054	0.7271	1.4036	0.0104	0.4247	1.6480	0.0006	0.2393	1.7084	0.0011	0.0350
CDP- Choline	Phospholipid Metabolism	1.0719	0.5737	0.7690	1.2820	0.1605	0.7109	1.4968	0.0104	0.7889	1.3742	0.0148	0.1605
1,2-dilinoleoyl-GPC (18:2/18:2)	Phosphatidylcholine	1.2919	0.0650	0.2519	1.1205	0.2786	0.8274	1.1483	0.2345	0.8477	1.4475	0.0104	0.1296
1,2-dipalmitoyl-GPC (16:0/16:0)	Phosphatidylcholine	0.8543	0.1605	0.4061	0.9775	0.7209	0.9796	0.8107	0.0830	0.7889	0.8351	0.2786	0.5573
1-linoleoyl-2-arachidonoyl-GPC (18:2/20:4n6)*	Phosphatidylcholine	1.3179	0.0019	0.0339	0.9339	0.7209	0.9796	1.0443	0.5737	0.9420	1.2309	0.0207	0.1834
1-linoleoyl-2-linolenoyl-GPC (18:2/18:3)*	Phosphatidylcholine	1.7076	0.0178	0.1387	0.9501	1.0000	1.0000	1.1991	0.2268	0.8477	1.6625	0.0074	0.1053
1-myristoyl-2-arachidonoyl-GPC (14:0/20:4)*	Phosphatidylcholine	1.5725	0.0070	0.0742	0.7966	0.1605	0.7109	0.7812	0.1605	0.8245	1.2526	0.0650	0.2974
1-myristoyl-2-palmitoyl-GPC (14:0/16:0)	Phosphatidylcholine	1.3765	0.1049	0.3231	0.8283	0.5054	0.9281	0.8263	0.3282	0.8684	1.1401	0.2786	0.5573
1-oleoyl-2-docosahexaenoyl-GPC (18:1/22:6)*	Phosphatidylcholine	1.0194	0.7209	0.8715	1.0108	0.8785	0.9796	0.9591	0.7209	0.9807	1.0304	0.5737	0.7690
1-oleoyl-2-linoleoyl-GPC (18:1/18:2)*	Phosphatidylcholine	1.1903	0.0830	0.2843	1.1775	0.0830	0.6305	1.1763	0.0207	0.7889	1.4015	0.0003	0.0185
1-palmitoleoyl-2-linolenoyl-GPC (16:1/18:3)*	Phosphatidylcholine	1.9314	0.0018	0.0339	0.9385	0.7209	0.9796	1.0140	0.7914	0.9868	1.8127	0.0095	0.1296
1-palmitoleoyl-2-linoleoyl-GPC (16:1/18:2)*	Phosphatidylcholine	1.5957	0.0006	0.0250	0.9692	1.0000	1.0000	1.1422	0.1605	0.8245	1.5466	0.0006	0.0253
1-palmitoyl-2-arachidonoyl-GPC (16:0/20:4n6)	Phosphatidylcholine	0.9960	0.7984	0.9201	0.9604	0.4418	0.8963	0.9605	0.4418	0.8905	0.9566	0.3823	0.6306
1-palmitoyl-2-dihomo-linolenoyl-GPC (16:0/20:3n3 or 6)*	Phosphatidylcholine	0.9335	0.8785	0.9629	1.0192	0.2786	0.8274	0.9306	0.3282	0.8684	0.9514	0.1605	0.4090
1-palmitoyl-2-docosahexaenoyl-GPC (16:0/22:6)	Phosphatidylcholine	0.9153	0.0830	0.2843	1.0216	0.7984	0.9796	0.9673	0.4418	0.8905	0.9350	0.1949	0.4504
1-palmitoyl-2-gamma-linolenoyl-GPC (16:0/18:3n6)*	Phosphatidylcholine	1.0843	0.6454	0.8232	0.9597	0.9591	0.9951	0.8708	0.5737	0.9420	1.0406	0.9591	0.9833
1-palmitoyl-2-linoleoyl-GPC (16:0/18:2)	Phosphatidylcholine	1.0093	0.9591	0.9849	1.0364	0.7984	0.9796	1.0178	0.6454	0.9802	1.0460	0.3823	0.6306
1-palmitoyl-2-oleoyl-GPC (16:0/18:1)	Phosphatidylcholine	1.0570	0.5737	0.7690	0.9610	0.6454	0.9796	0.9391	0.4418	0.8905	1.0158	1.0000	1.0000
1-palmitoyl-2-palmitoleoyl-GPC (16:0/16:1)*	Phosphatidylcholine	1.1567	0.3282	0.6013	0.8960	0.2345	0.7971	0.9148	0.7984	0.9868	1.0364	0.7984	0.9012
1-palmitoyl-2-stearoyl-GPC (16:0/18:0)	Phosphatidylcholine	1.1499	0.5737	0.7690	0.9598	0.7984	0.9796	0.8540	0.5054	0.9265	1.1036	0.7209	0.8497
1-stearoyl-2-arachidonoyl-GPC (18:0/20:4)	Phosphatidylcholine	1.2003	0.0070	0.0742	0.8845	0.0379	0.6304	0.9680	0.4418	0.8905	1.0617	0.5054	0.7211
1-stearoyl-2-docosahexaenoyl-GPC (18:0/22:6)	Phosphatidylcholine	1.1949	0.0148	0.1163	0.8843	0.1949	0.7477	0.9492	0.5054	0.9265	1.0566	0.7984	0.9012
1-stearoyl-2-linoleoyl-GPC (18:0/18:2)*	Phosphatidylcholine	1.3288	0.0019	0.0339	0.9537	0.6454	0.9796	1.0638	1.0000	1.0000	1.2672	0.0070	0.1038
1-stearoyl-2-oleoyl-GPC (18:0/18:1)	Phosphatidylcholine	1.8256	0.0030	0.0442	0.8762	0.6454	0.9796	1.1013	0.8785	0.9992	1.5996	0.0047	0.0837
ceramide (d18:1/14:0, d16:1/16:0)*	Ceramides	1.3196	0.1304	0.3583	0.9801	0.8785	0.9796	0.9447	0.4418	0.8905	1.2934	0.0830	0.3110
ceramide (d18:1/17:0, d17:1/18:0)*	Ceramides	1.2715	0.4619	0.7217	1.0711	0.7525	0.9796	0.9485	0.6742	0.9807	1.3523	0.1880	0.4504
ceramide (d18:2/24:1, d18:1/24:2)*	Ceramides	1.7711	0.0070	0.0742	0.9251	0.9591	0.9951	1.0097	0.9591	1.0000	1.6383	0.0281	0.2011
N-palmitoyl-sphingadienine (d18:2/16:0)*	Ceramides	1.1326	0.2345	0.5061	0.9638	0.5054	0.9281	0.8270	0.1304	0.8245	1.0916	0.2345	0.5071
N-palmitoyl-sphingosine (d18:1/16:0)	Ceramides	1.4685	0.0830	0.2843	1.0025	0.7984	0.9796	0.9813	0.7209	0.9807	1.4721	0.1605	0.4090
N-stearoyl-sphingosine (d18:1/18:0)*	Ceramides	1.3050	0.2786	0.5587	1.0172	1.0000	1.0000	1.0299	0.6454	0.9802	1.3274	0.3823	0.6306
N-palmitoyl-sphinganine (d18:0/16:0)	Ceramides	1.1263	0.6454	0.8232	1.3031	0.3823	0.8514	0.9656	0.2786	0.8651	1.4678	0.7209	0.8497
behenoyl sphingomyelin (d18:1/22:0)*	Sphingomyelins	1.3257	0.3282	0.6013	0.9524	0.9591	0.9951	0.9123	0.7209	0.9807	1.2626	0.5054	0.7211
lignoceroyl sphingomyelin (d18:1/24:0)	Sphingomyelins	1.3701	0.2345	0.5061	0.9303	0.9591	0.9951	0.8943	0.7209	0.9807	1.2746	0.3823	0.6306
palmitoyl sphingomyelin (d18:1/16:0)	Sphingomyelins	0.9816	0.8785	0.9629	0.9105	0.5054	0.9281	0.8867	0.2786	0.8651	0.8938	0.3282	0.5879
sphingomyelin (d17:1/16:0, d18:1/15:0, d16:1/17:0)*	Sphingomyelins	1.0672	0.4418	0.6974	0.8925	0.5737	0.9583	0.9196	0.2786	0.8651	0.9525	0.4418	0.6714
sphingomyelin (d18:1/14:0, d16:1/16:0)*	Sphingomyelins	1.1706	0.0379	0.1944	0.9217	0.5737	0.9583	1.0345	1.0000	1.0000	1.0790	0.5054	0.7211
sphingomyelin (d18:1/17:0, d17:1/18:0, d19:1/16:0)	Sphingomyelins	1.0561	0.9591	0.9849	0.8674	0.3823	0.8514	0.8619	0.5737	0.9420	0.9161	0.8785	0.9419
sphingomyelin (d18:1/18:1, d18:2/18:0)	Sphingomyelins	0.9325	0.3282	0.6013	0.9106	0.1605	0.7109	0.9644	0.4418	0.8905	0.8491	0.0207	0.1834
sphingomyelin (d18:1/19:0, d19:1/18:0)*	Sphingomyelins	1.2845	0.3282	0.6013	0.8563	0.5737	0.9583	0.8432	0.6454	0.9802	1.1000	0.5737	0.7690
sphingomyelin (d18:1/20:0, d16:1/22:0)*	Sphingomyelins	1.3274	0.1605	0.4061	0.8818	0.7209	0.9796	0.8968	0.5054	0.9265	1.1705	0.5054	0.7211
sphingomyelin (d18:1/20:1, d18:2/20:0)*	Sphingomyelins	0.8699	0.1949	0.4553	0.9227	0.2345	0.7971	0.8970	0.2786	0.8651	0.8027	0.1049	0.3201
sphingomyelin (d18:1/21:0, d17:1/22:0, d16:1/23:0)*	Sphingomyelins	1.2710	0.2786	0.5587	0.9486	0.9591	0.9951	0.8816	0.4418	0.8905	1.2056	0.4418	0.6714
sphingomyelin (d18:1/22:1, d18:2/22:0, d16:1/24:1)*	Sphingomyelins	1.1856	0.2345	0.5061	0.8689	0.4418	0.8963	0.8483	0.3823	0.8787	1.0302	0.6454	0.8115
sphingomyelin (d18:1/22:2, d18:2/22:1, d16:1/24:2)*	Sphingomyelins	0.9755	0.9591	0.9849	0.9596	0.3823	0.8514	1.0118	0.7984	0.9868	0.9361	0.7209	0.8497
sphingomyelin (d18:1/24:1, d18:2/24:0)*	Sphingomyelins	1.3253	0.1949	0.4553	0.8337	0.4418	0.8963	0.8408	0.4418	0.8905	1.1049	0.5054	0.7211
sphingomyelin (d18:2/16:0, d18:1/16:1)*	Sphingomyelins	1.0627	0.5054	0.7271	0.9212	0.3282	0.8395	1.0010	0.8785	0.9992	0.9790	0.6454	0.8115
sphingomyelin (d18:2/23:0, d18:1/23:1, d17:1/24:1)*	Sphingomyelins	1.1710	0.3823	0.6490	0.8636	0.3282	0.8395	0.8267	0.5054	0.9265	1.0113	0.8785	0.9419
sphingomyelin (d18:2/24:1, d18:1/24:2)*	Sphingomyelins	1.1420	0.5054	0.7271	0.8648	0.4418	0.8963	0.8302	0.5737	0.9420	0.9876	1.0000	1.0000
stearoyl sphingomyelin (d18:1/18:0)	Sphingomyelins	1.1136	0.3823	0.6490	0.8496	0.2345	0.7971	0.9162	0.4418	0.8905	0.9462	0.7209	0.8497
tricosanoyl sphingomyelin (d18:1/23:0)*	Sphingomyelins	1.3825	0.5737	0.7690	0.9915	0.9591	0.9951	0.7118	0.5054	0.9265	1.3707	0.5054	0.7211
behenoyl dihydrosphingomyelin (d18:0/22:0)*	Sphingomyelins	1.8982	0.1690	0.4261	0.8745	0.9163	0.9951	0.9660	0.9141	1.0000	1.6659	0.1550	0.4090
palmitoyl dihydrosphingomyelin (d18:0/16:0)*	Sphingomyelins	1.0743	0.7209	0.8715	1.0318	0.7209	0.9796	0.9935	0.9591	1.0000	1.1084	1.0000	1.0000
diacylglycerol (16:1/18:2 [2], 16:0/18:3 [1])*	Diacylglycerols	1.3870	0.0499	0.2203	0.9924	1.0000	1.0000	1.0950	0.3282	0.8684	1.3765	0.0379	0.2342
linoleoyl-arachidonoyl-glycerol (18:2/20:4) [2]*	Diacylglycerols	0.9222	0.4418	0.6974	1.3627	0.1049	0.7109	1.4423	0.0650	0.7889	1.2566	0.2786	0.5573
linoleoyl-docosahexaenoyl-glycerol (18:2/22:6) [1]*	Diacylglycerols	1.3978	0.1149	0.3526	1.2439	0.5737	0.9583	1.6278	0.0100	0.7889	1.7299	0.1033	0.3201
linoleoyl-docosahexaenoyl-glycerol (18:2/22:6) [2]*	Diacylglycerols	0.9775	0.8785	0.9629	1.4076	0.2345	0.7971	1.3001	0.0499	0.7889	1.3759	0.4418	0.6714

linoleoyl-linolenoyl-glycerol (18:2/18:3) [2]*	Diacylglycerols	1.0001	0.9591	0.9849	1.2317	0.5054	0.9281	1.2009	0.2786	0.8651	1.2318	0.4418	0.6714
linoleoyl-linoleoyl-glycerol (18:2/18:2) [1]*	Diacylglycerols	1.0432	0.7984	0.9201	1.3337	0.5737	0.9583	1.3950	0.2345	0.8477	1.3913	0.3282	0.5879
linoleoyl-linoleoyl-glycerol (18:2/18:2) [2]*	Diacylglycerols	0.7426	0.1949	0.4553	1.6088	0.2786	0.8274	1.2943	0.1605	0.8245	1.1948	0.8785	0.9419
oleoyl-arachidonoyl-glycerol (18:1/20:4) [1]*	Diacylglycerols	1.6185	0.0030	0.0442	0.9828	0.7209	0.9796	1.2521	0.3282	0.8684	1.5907	0.0281	0.2011
oleoyl-arachidonoyl-glycerol (18:1/20:4) [2]*	Diacylglycerols	1.1958	0.4418	0.6974	1.0690	0.7209	0.9796	1.1416	0.2786	0.8651	1.2783	0.0830	0.3110
oleoyl-linoleoyl-glycerol (18:1/18:2) [1]	Diacylglycerols	1.2933	0.1049	0.3231	1.1813	0.8785	0.9796	1.3392	0.1049	0.8245	1.5277	0.0650	0.2974
oleoyl-linoleoyl-glycerol (18:1/18:2) [2]	Diacylglycerols	1.0477	0.5737	0.7690	1.2263	0.3282	0.8395	1.1836	0.1049	0.8245	1.2847	0.3282	0.5879
oleoyl-oleoyl-glycerol (18:1/18:1) [1]*	Diacylglycerols	1.7714	0.0519	0.2238	1.0503	0.9591	0.9951	1.3238	0.2687	0.8651	1.8606	0.1559	0.4090
oleoyl-oleoyl-glycerol (18:1/18:1) [2]*	Diacylglycerols	1.2429	0.1304	0.3583	1.1534	0.6454	0.9796	1.0839	0.7209	0.9807	1.4335	0.0650	0.2974
palmitoleoyl-arachidonoyl-glycerol (16:1/20:4) [2]*	Diacylglycerols	0.7872	0.1304	0.3583	1.2293	0.2345	0.7971	1.0998	0.6454	0.9802	0.9677	0.9591	0.9833
palmitoleoyl-linoleoyl-glycerol (16:1/18:2) [1]*	Diacylglycerols	1.8401	0.0047	0.0594	0.9510	0.7209	0.9796	1.3960	0.0650	0.7889	1.7500	0.0104	0.1296
palmitoyl-arachidonoyl-glycerol (16:0/20:4) [2]*	Diacylglycerols	0.9666	0.7209	0.8715	0.9153	0.7984	0.9796	0.8176	0.2786	0.8651	0.8847	0.5054	0.7211
palmitoyl-docosahexaenoyl-glycerol (16:0/22:6) [1]*	Diacylglycerols	1.1093	0.3282	0.6013	0.6733	0.0650	0.6304	0.6661	0.1304	0.8245	0.7468	0.3823	0.6306
palmitoyl-docosahexaenoyl-glycerol (16:0/22:6) [2]*	Diacylglycerols	0.8686	0.4418	0.6974	0.8483	0.7984	0.9796	0.5365	0.0104	0.7889	0.7369	0.1949	0.4504
palmitoyl-linolenoyl-glycerol (16:0/18:3) [2]*	Diacylglycerols	1.2975	0.1880	0.4553	0.9104	0.4005	0.8792	0.9588	1.0000	1.0000	1.1923	0.7130	0.8497
palmitoyl-linoleoyl-glycerol (16:0/18:2) [1]*	Diacylglycerols	1.1681	0.3823	0.6490	0.9767	0.7209	0.9796	1.0121	1.0000	1.0000	1.1409	0.8785	0.9419
palmitoyl-linoleoyl-glycerol (16:0/18:2) [2]*	Diacylglycerols	0.9813	0.9591	0.9849	1.0445	0.9591	0.9951	0.9113	0.4418	0.8905	1.0250	0.7984	0.9012
palmitoyl-oleoyl-glycerol (16:0/18:1) [2]*	Diacylglycerols	1.1958	0.5737	0.7690	0.8975	0.8785	0.9796	0.7640	0.1049	0.8245	1.0732	0.9591	0.9833
stearoyl-arachidonoyl-glycerol (18:0/20:4) [2]*	Diacylglycerols	1.2729	0.2786	0.5587	0.9734	1.0000	1.0000	1.1198	0.7209	0.9807	1.2389	0.3823	0.6306
stearoyl-docosahexaenoyl-glycerol (18:0/22:6) [2]*	Diacylglycerols	1.4905	0.1304	0.3583	0.9246	0.9591	0.9951	0.8747	0.4418	0.8905	1.3782	0.4418	0.6714
stearoyl-linoleoyl-glycerol (18:0/18:2) [2]*	Diacylglycerols	1.2419	0.4619	0.7217	1.2502	0.6742	0.9796	1.1134	0.6355	0.9802	1.5479	0.3170	0.5879
PEMT Pathway													
CDP- Ethanolamine	Phospholipid Metabolism	0.9994	1.0000	1.0000	0.9225	0.9591	0.9951	1.1855	0.0650	0.7889	0.9220	0.8785	0.9419
1,2-dipalmitoyl-GPE (16:0/16:0)*	Phosphatidylethanolamines	1.0368	0.9591	0.9849	0.8290	0.8745	0.9796	0.6375	0.1889	0.8245	0.8757	0.6355	0.8115
1-linoleoyl-2-arachidonoyl-GPE (18:2/20:4)*	Phosphatidylethanolamines	1.6771	0.0003	0.0183	0.7705	0.1949	0.7477	0.9499	0.5737	0.9420	1.2922	0.0281	0.2011
1-oleoyl-2-arachidonoyl-GPE (18:1/20:4)*	Phosphatidylethanolamines	1.4426	0.0003	0.0183	0.8436	0.1949	0.7477	0.9215	0.1049	0.8245	1.2171	0.0148	0.1605
1-oleoyl-2-docosahexaenoyl-GPE (18:1/22:6)*	Phosphatidylethanolamines	1.5084	0.0006	0.0250	0.8345	0.0281	0.6304	0.9013	0.3282	0.8684	1.2588	0.0207	0.1834
1-oleoyl-2-linoleoyl-GPE (18:1/18:2)*	Phosphatidylethanolamines	1.3872	0.0379	0.1944	0.8151	0.3282	0.8395	0.8884	0.5737	0.9420	1.1307	0.2786	0.5573
1-palmitoyl-2-arachidonoyl-GPE (16:0/20:4)*	Phosphatidylethanolamines	1.1242	0.3823	0.6490	0.9428	0.6454	0.9796	0.9504	0.7209	0.9807	1.0599	0.7984	0.9012
1-palmitoyl-2-docosahexaenoyl-GPE (16:0/22:6)*	Phosphatidylethanolamines	1.1063	0.4418	0.6974	0.9466	0.5054	0.9281	0.9425	0.8785	0.9992	1.0472	0.7209	0.8497
1-palmitoyl-2-linoleoyl-GPE (16:0/18:2)	Phosphatidylethanolamines	1.2749	0.3823	0.6490	0.8254	0.3823	0.8514	0.9387	0.7984	0.9868	1.0523	0.6454	0.8115
1-palmitoyl-2-oleoyl-GPE (16:0/18:1)	Phosphatidylethanolamines	1.3840	0.2345	0.5061	0.7485	0.3823	0.8514	0.7908	0.3282	0.8684	1.0359	0.6454	0.8115
1-stearoyl-2-arachidonoyl-GPE (18:0/20:4)	Phosphatidylethanolamines	1.4000	0.0281	0.1692	0.8995	0.5737	0.9583	1.0311	1.0000	1.0000	1.2593	0.1049	0.3201
1-stearoyl-2-docosahexaenoyl-GPE (18:0/22:6)*	Phosphatidylethanolamines	1.4387	0.0650	0.2519	0.8555	0.4418	0.8963	0.9091	1.0000	1.0000	1.2308	0.3282	0.5879
1-stearoyl-2-linoleoyl-GPE (18:0/18:2)*	Phosphatidylethanolamines	1.5886	0.0379	0.1944	0.8496	0.4418	0.8963	1.0393	1.0000	1.0000	1.3496	0.1949	0.4504
1-stearoyl-2-oleoyl-GPE (18:0/18:1)	Phosphatidylethanolamines	1.8756	0.0499	0.2203	0.8406	0.6454	0.9796	0.9510	1.0000	1.0000	1.5767	0.1605	0.4090
Methyl Donor Pathway													
Betaine	Glycine, Serine and Threonine Metabolism	1.0347	0.9591	0.9849	1.5433	0.0379	0.6304	1.4376	0.0650	0.7889	1.5968	0.0207	0.1834
Dimethylglycine	Glycine, Serine and Threonine Metabolism	0.7121	0.0104	0.0970	1.6997	0.0003	0.1204	1.1436	0.1949	0.8245	1.2103	0.1049	0.3201
Sarcosine	Glycine, Serine and Threonine Metabolism	0.8742	0.5737	0.7690	1.7170	0.1949	0.7477	1.2436	0.3282	0.8684	1.5010	0.4418	0.6714
Methionine	Methionine, Cysteine, SAM and Taurine Metabolism	0.8760	0.7209	0.8715	1.0273	0.8785	0.9796	0.7627	0.3282	0.8684	0.8999	0.8785	0.9419
Cysteine	Methionine, Cysteine, SAM and Taurine Metabolism	0.9693	0.5054	0.7271	1.0639	0.3282	0.8395	1.0671	0.5054	0.9265	1.0312	0.7209	0.8497
Other Metabolites													
S-adenosylmethionine (SAM)	Methionine, Cysteine, SAM and Taurine Metabolism	0.7289	0.0104	0.0970	1.2648	0.0379	0.6304	0.9716	0.2345	0.8477	0.9219	0.1605	0.4090
S-adenosylhomocysteine (SAH)	Methionine, Cysteine, SAM and Taurine Metabolism	0.9832	1.0000	1.0000	1.1217	0.1949	0.7477	0.9501	0.5737	0.9420	1.1028	0.3282	0.5879
Serine	Glycine, Serine and Threonine Metabolism	0.8944	0.3823	0.6490	1.1528	0.2786	0.8274	0.9190	0.5737	0.9420	1.0311	0.8785	0.9419
Glycine	Glycine, Serine and Threonine Metabolism	1.1310	0.5737	0.7690	0.9828	0.9591	0.9951	1.0114	0.9591	1.0000	1.1116	0.5737	0.7690
trimethylamine N-oxide (TMAO)	Phospholipid Metabolism	0.2697	0.0070	0.0742	2.4409	0.0281	0.6304	0.4413	0.1304	0.8245	0.6584	0.3823	0.6306
**PLACENTA**													
Choline	Phospholipid Metabolism	1.0104	0.6454	0.8466	1.0262	0.2810	0.5785	0.9713	0.7984	0.9611	1.0356	0.5054	0.7473
CDP-Choline Pathway													
Phosphocholine	Phospholipid Metabolism	1.1312	0.0379	0.3037	0.9133	0.1206	0.4065	1.0204	0.5737	0.8705	1.0518	0.2786	0.5952
CDP- Choline	Phospholipid Metabolism	1.0790	0.3823	0.6968	0.8862	0.2810	0.5785	1.0048	1.0000	1.0000	0.9437	0.6454	0.8472
1,2-dilinoleoyl-GPC (18:2/18:2)	Phosphatidylcholine	0.9716	1.0000	1.0000	1.0031	0.8665	0.9482	0.9774	0.8785	0.9849	0.9692	0.8785	0.9420
1,2-dipalmitoyl-GPC (16:0/16:0)	Phosphatidylcholine	1.0837	0.1605	0.5265	1.0103	0.9551	0.9773	1.1316	0.0650	0.5438	1.0761	0.3823	0.6591
1-linoleoyl-2-arachidonoyl-GPC (18:2/20:4n6)*	Phosphatidylcholine	1.1287	0.0207	0.2265	0.9042	0.0401	0.2660	1.0114	0.9591	0.9900	1.0215	0.3823	0.6591
1-myristoyl-2-arachidonoyl-GPC (14:0/20:4)*	Phosphatidylcholine	1.3551	0.0030	0.0703	0.8944	0.0401	0.2660	1.0912	0.2345	0.7129	1.2046	0.0104	0.1291
1-myristoyl-2-palmitoyl-GPC (14:0/16:0)	Phosphatidylcholine	1.2804	0.0011	0.0431	0.9178	0.1893	0.4884	1.1146	0.0379	0.5264	1.1805	0.0104	0.1291
1-oleoyl-2-docosahexaenoyl-GPC (18:1/22:6)*	Phosphatidylcholine	1.0759	0.5054	0.7781	1.0640	0.3969	0.6744	1.2428	0.0070	0.3236	1.1399	0.1949	0.4967
1-oleoyl-2-linoleoyl-GPC (18:1/18:2)*	Phosphatidylcholine	1.1578	0.0650	0.3650	0.9697	0.6126	0.8341	1.1202	0.1304	0.6067	1.1086	0.3282	0.6270
1-palmitoyl-2-arachidonoyl-GPC (16:0/20:4n6)	Phosphatidylcholine	1.1043	0.0104	0.1522	0.9523	0.1520	0.4587	1.0380	0.3282	0.7532	1.0378	0.1605	0.4584
1-palmitoyl-2-dihomo-linolenoyl-GPC (16:0/20:3n3 or 6)*	Phosphatidylcholine	1.1568	0.0650	0.3650	0.8340	0.0939	0.3809	1.0903	0.2786	0.7368	0.9687	0.3823	0.6591
1-palmitoyl-2-docosahexaenoyl-GPC (16:0/22:6)	Phosphatidylcholine	1.0879	0.1049	0.4391	1.0209	0.7789	0.9119	1.1309	0.0379	0.5264	1.0879	0.1605	0.4584
1-palmitoyl-2-gamma-linolenoyl-GPC (16:0/18:3n6)*	Phosphatidylcholine	0.9119	0.5737	0.8046	0.9492	0.7789	0.9119	0.8792	0.5737	0.8705	0.8451	0.3282	0.6270
1-palmitoyl-2-linoleoyl-GPC (16:0/18:2)	Phosphatidylcholine	1.0693	0.0499	0.3463	0.9685	0.5358	0.7818	1.0773	0.0379	0.5264	1.0211	0.6454	0.8472
1-palmitoyl-2-oleoyl-GPC (16:0/18:1)	Phosphatidylcholine	1.1403	0.0070	0.1239	0.9330	0.1520	0.4587	1.1489	0.0019	0.1553	1.0605	0.1949	0.4967
1-palmitoyl-2-palmitoleoyl-GPC (16:0/16:1)*	Phosphatidylcholine	1.1556	0.0104	0.1522	0.9462	0.3969	0.6744	1.1006	0.1304	0.6067	1.1017	0.0281	0.2184
1-palmitoyl-2-stearoyl-GPC (16:0/18:0)	Phosphatidylcholine	1.1357	0.1949	0.5617	0.9585	0.6943	0.8768	1.0839	0.1605	0.6249	1.0646	0.3282	0.6270
1-stearoyl-2-arachidonoyl-GPC (18:0/20:4)	Phosphatidylcholine	1.1982	0.0019	0.0597	0.9128	0.0721	0.3464	1.0629	0.0379	0.5264	1.0915	0.0030	0.0791
1-stearoyl-2-docosahexaenoyl-GPC (18:0/22:6)	Phosphatidylcholine	1.1510	0.0104	0.1522	0.9832	0.3357	0.6250	1.1481	0.0104	0.3469	1.1269	0.0047	0.0945
1-stearoyl-2-linoleoyl-GPC (18:0/18:2)*	Phosphatidylcholine	1.1808	0.0070	0.1239	0.9294	0.1520	0.4587	1.0928	0.0281	0.5264	1.0891	0.0830	0.3555
1-stearoyl-2-oleoyl-GPC (18:0/18:1)	Phosphatidylcholine	1.2427	0.0030	0.0703	0.9052	0.0939	0.3809	1.1813	0.0030	0.2050	1.1223	0.0148	0.1573
ceramide (d18:1/17:0, d17:1/18:0)*	Ceramides	1.0847	0.7899	0.9136	0.8210	0.4437	0.7282	1.0228	0.8730	0.9849	0.8695	0.5577	0.8040
ceramide (d18:2/24:1, d18:1/24:2)*	Ceramides	1.0217	0.7209	0.8792	0.9734	0.3969	0.6744	1.1393	0.1049	0.6067	0.9792	0.7209	0.8810
N-(2-hydroxypalmitoyl)-sphingosine (d18:1/16:0(2OH))	Ceramides	1.3610	0.0650	0.3650	0.7472	0.3357	0.6250	1.1810	0.1605	0.6249	0.9677	0.7984	0.9052
N-behenoyl-sphingadienine (d18:2/22:0)*	Ceramides	0.9774	0.7209	0.8792	1.1879	0.1206	0.4065	1.2220	0.1049	0.6067	1.1133	0.3823	0.6591
N-palmitoyl-sphingadienine (d18:2/16:0)*	Ceramides	0.9848	0.9591	0.9779	0.9866	0.8665	0.9482	1.0837	0.2786	0.7368	0.9534	0.5054	0.7473
N-palmitoyl-sphingosine (d18:1/16:0)	Ceramides	1.1520	0.0379	0.3037	0.9244	0.2810	0.5785	1.0909	0.0499	0.5327	1.0304	0.8785	0.9420
N-stearoyl-sphingosine (d18:1/18:0)*	Ceramides	1.0392	0.9591	0.9779	1.0607	0.5358	0.7818	1.1045	0.5054	0.8419	1.0684	0.7209	0.8810

N-palmitoyl-sphinganine (d18:0/16:0)	Ceramides	1.1507	0.3823	0.6968	0.8331	0.1206	0.4065	0.9472	0.9591	0.9900	0.9456	0.7984	0.9052
behenoyl sphingomyelin (d18:1/22:0)*	Sphingomyelins	1.2310	0.1304	0.4722	1.1313	0.2810	0.5785	1.2638	0.0830	0.5809	1.3527	0.0104	0.1291
hydroxypalmitoyl sphingomyelin (d18:1/16:0(OH))	Sphingomyelins	1.0644	0.5624	0.8046	1.0349	0.4835	0.7584	1.1655	0.0499	0.5327	1.0922	0.3442	0.6531
lignoceroyl sphingomyelin (d18:1/24:0)	Sphingomyelins	1.0866	0.2786	0.6307	1.0229	0.9551	0.9773	1.0895	0.1605	0.6249	1.0936	0.1949	0.4967
palmitoyl sphingomyelin (d18:1/16:0)	Sphingomyelins	1.0814	0.1304	0.4722	0.9391	0.1893	0.4884	1.0406	0.2786	0.7368	1.0160	0.4418	0.7033
sphingomyelin (d17:1/14:0, d16:1/15:0)*	Sphingomyelins	1.0038	0.9591	0.9779	1.0972	0.3969	0.6744	1.1344	0.0379	0.5264	1.0627	0.8785	0.9420
sphingomyelin (d17:1/16:0, d18:1/15:0, d16:1/17:0)*	Sphingomyelins	1.1181	0.0830	0.3996	0.9404	0.1893	0.4884	1.0661	0.3282	0.7532	1.0567	0.6454	0.8472
sphingomyelin (d17:2/16:0, d18:2/15:0)*	Sphingomyelins	0.8265	0.5898	0.8243	1.1563	0.1611	0.4845	0.8856	0.6314	0.9020	0.9199	1.0000	1.0000
sphingomyelin (d18:1/14:0, d16:1/16:0)*	Sphingomyelins	1.1552	0.0207	0.2265	0.9291	0.1893	0.4884	1.1161	0.0650	0.5438	1.0780	0.1049	0.3891
sphingomyelin (d18:1/17:0, d17:1/18:0, d19:1/16:0)	Sphingomyelins	1.1999	0.0030	0.0703	0.8871	0.1206	0.4065	1.0564	0.4418	0.8088	1.0749	0.2345	0.5324
sphingomyelin (d18:1/18:1, d18:2/18:0)	Sphingomyelins	1.1440	0.0047	0.0903	0.8665	0.0059	0.2146	1.0235	0.3823	0.7673	0.9974	1.0000	1.0000
sphingomyelin (d18:1/19:0, d19:1/18:0)*	Sphingomyelins	1.3254	0.0927	0.4391	1.1191	0.6022	0.8341	1.3113	0.0458	0.5327	1.5078	0.0047	0.0945
sphingomyelin (d18:1/20:0, d16:1/22:0)*	Sphingomyelins	1.1849	0.1304	0.4722	1.1273	0.3357	0.6250	1.1763	0.1304	0.6067	1.2909	0.0148	0.1573
sphingomyelin (d18:1/20:1, d18:2/20:0)*	Sphingomyelins	1.0918	0.2345	0.6029	0.9884	0.7789	0.9119	1.0917	0.1949	0.6878	1.0816	0.3282	0.6270
sphingomyelin (d18:1/21:0, d17:1/22:0, d16:1/23:0)*	Sphingomyelins	1.1365	0.2345	0.6029	1.2340	0.1520	0.4587	1.2617	0.0650	0.5438	1.3752	0.0207	0.1908
sphingomyelin (d18:1/22:1, d18:2/22:0, d16:1/24:1)*	Sphingomyelins	1.1529	0.1049	0.4391	1.0338	0.7789	0.9119	1.1725	0.0019	0.1553	1.1676	0.0070	0.1118
sphingomyelin (d18:1/22:2, d18:2/22:1, d16:1/24:2)*	Sphingomyelins	1.0999	0.1049	0.4391	0.9662	0.4634	0.7282	1.1466	0.0207	0.4919	1.0840	0.2345	0.5324
sphingomyelin (d18:1/24:1, d18:2/24:0)*	Sphingomyelins	1.2353	0.0104	0.1522	0.9885	0.8665	0.9482	1.1895	0.0047	0.2589	1.2092	0.0070	0.1118
sphingomyelin (d18:2/16:0, d18:1/16:1)*	Sphingomyelins	1.0822	0.2786	0.6307	0.8040	0.0401	0.2660	1.0304	0.7984	0.9611	0.8599	0.0207	0.1908
sphingomyelin (d18:2/18:1)*	Sphingomyelins	1.1781	0.1949	0.5617	0.9284	0.6943	0.8768	1.1899	0.1304	0.6067	1.1178	0.4418	0.7033
sphingomyelin (d18:2/23:0, d18:1/23:1, d17:1/24:1)*	Sphingomyelins	1.1283	0.1605	0.5265	1.0228	1.0000	1.0000	1.2234	0.0047	0.2589	1.1467	0.0207	0.1908
sphingomyelin (d18:2/24:1, d18:1/24:2)*	Sphingomyelins	1.1685	0.0281	0.2693	0.9416	0.3357	0.6250	1.2088	0.0006	0.1036	1.0941	0.1304	0.4282
sphingomyelin (d18:2/24:2)*	Sphingomyelins	1.0836	0.2345	0.6029	0.8738	0.0939	0.3809	1.1250	0.0499	0.5327	0.9582	0.6454	0.8472
stearoyl sphingomyelin (d18:1/18:0)	Sphingomyelins	1.2226	0.0030	0.0703	0.9815	0.6943	0.8768	1.1096	0.0379	0.5264	1.1843	0.0011	0.0452
tricosanoyl sphingomyelin (d18:1/23:0)*	Sphingomyelins	1.1269	0.2786	0.6307	1.1106	0.5358	0.7818	1.1689	0.0499	0.5327	1.2304	0.0650	0.3083
behenoyl dihydrosphingomyelin (d18:0/22:0)*	Sphingomyelins	1.1461	0.5737	0.8046	1.2872	0.0939	0.3809	1.2015	0.1949	0.6878	1.3878	0.1304	0.4282
palmitoyl dihydrosphingomyelin (d18:0/16:0)*	Sphingomyelins	1.1357	0.0281	0.2693	1.0429	0.4634	0.7282	1.1339	0.1049	0.6067	1.1701	0.0379	0.2521
sphingomyelin (d18:0/18:0, d19:0/17:0)*	Sphingomyelins	1.3723	0.0379	0.3037	1.1493	0.3969	0.6744	1.2710	0.1049	0.6067	1.5237	0.0148	0.1573
sphingomyelin (d18:0/20:0, d16:0/22:0)*	Sphingomyelins	1.2265	0.5283	0.8046	1.4971	0.0930	0.3809	1.2928	0.3442	0.7673	1.8262	0.0379	0.2521
diacylglycerol (16:1/18:2 [2], 16:0/18:3 [1])*	Diacylglycerols	1.5126	0.0379	0.3037	0.9964	0.7789	0.9119	1.3832	0.0148	0.4392	1.4496	0.0379	0.2521
linoleoyl-arachidonoyl-glycerol (18:2/20:4) [1]*	Diacylglycerols	1.3310	0.0653	0.3650	1.1713	0.3850	0.6744	1.3760	0.0653	0.5438	1.4972	0.0653	0.3083
linoleoyl-arachidonoyl-glycerol (18:2/20:4) [2]*	Diacylglycerols	1.2891	0.1304	0.4722	0.9273	0.4634	0.7282	1.0912	0.1304	0.6067	1.2217	0.0379	0.2521
linoleoyl-linoleoyl-glycerol (18:2/18:2) [1]*	Diacylglycerols	1.1863	0.1605	0.5265	1.0290	0.9551	0.9773	1.1541	0.3282	0.7532	1.2012	0.1949	0.4967
myristoyl-linoleoyl-glycerol (14:0/18:2) [2]*	Diacylglycerols	1.6299	0.0379	0.3037	0.9679	0.8665	0.9482	1.4872	0.0281	0.5264	1.5687	0.0499	0.2801
oleoyl-arachidonoyl-glycerol (18:1/20:4) [1]*	Diacylglycerols	1.1353	0.5737	0.8046	1.0418	0.8665	0.9482	1.0523	0.6454	0.9020	1.1263	0.6454	0.8472
oleoyl-arachidonoyl-glycerol (18:1/20:4) [2]*	Diacylglycerols	1.1174	0.1605	0.5265	0.9769	0.7209	0.8783	1.0719	0.4418	0.8088	1.0916	0.2786	0.5952
oleoyl-linoleoyl-glycerol (18:1/18:2) [2]	Diacylglycerols	1.3400	0.0650	0.3650	0.9585	0.5737	0.7898	1.2722	0.0379	0.5264	1.2844	0.0379	0.2521
palmitoyl-arachidonoyl-glycerol (16:0/20:4) [1]*	Diacylglycerols	0.9538	0.6454	0.8466	1.0801	0.6454	0.8247	0.9979	0.8785	0.9849	1.0302	0.8785	0.9420
palmitoyl-arachidonoyl-glycerol (16:0/20:4) [2]*	Diacylglycerols	0.9937	0.9591	0.9779	1.0583	0.7209	0.8783	1.0635	0.3823	0.7673	1.0517	0.5737	0.8040
palmitoyl-dihomo-linolenoyl-glycerol (16:0/20:3n3 or 6) [2]*	Diacylglycerols	1.0768	0.4834	0.7781	0.9062	0.4834	0.7526	1.0724	0.6314	0.9020	0.9771	0.7012	0.8810
palmitoyl-docosahexaenoyl-glycerol (16:0/22:6) [1]*	Diacylglycerols	1.0922	0.5737	0.8046	1.0207	0.7984	0.9360	1.1579	0.1605	0.6249	1.1148	0.6454	0.8472
palmitoyl-docosahexaenoyl-glycerol (16:0/22:6) [2]*	Diacylglycerols	1.0865	0.8785	0.9504	1.0384	1.0000	1.0000	1.2397	0.2786	0.7368	1.1282	0.5054	0.7473
palmitoyl-linoleoyl-glycerol (16:0/18:2) [1]*	Diacylglycerols	1.4090	0.2575	0.6307	1.0871	0.6314	0.8247	1.4070	0.3112	0.7532	1.5369	0.1455	0.4584
palmitoyl-linoleoyl-glycerol (16:0/18:2) [2]*	Diacylglycerols	1.1645	0.1605	0.5265	1.0338	0.8785	0.9637	1.2232	0.0379	0.5264	1.2039	0.0499	0.2801
stearoyl-arachidonoyl-glycerol (18:0/20:4) [1]*	Diacylglycerols	1.0260	0.7209	0.8792	0.8449	0.3823	0.6638	0.9237	0.9591	0.9900	0.8668	0.5054	0.7473
stearoyl-arachidonoyl-glycerol (18:0/20:4) [2]*	Diacylglycerols	1.0432	0.6454	0.8466	0.9431	0.4418	0.7111	1.0537	0.4418	0.8088	0.9838	0.8785	0.9420
stearoyl-docosahexaenoyl-glycerol (18:0/22:6) [1]*	Diacylglycerols	1.0540	0.8785	0.9504	1.0214	0.6454	0.8247	1.0554	0.5737	0.8705	1.0766	0.7209	0.8810
stearoyl-docosahexaenoyl-glycerol (18:0/22:6) [2]*	Diacylglycerols	1.0514	0.8785	0.9504	1.0300	0.7984	0.9360	1.1373	0.1949	0.6878	1.0829	0.5054	0.7473
stearoyl-linoleoyl-glycerol (18:0/18:2) [2]*	Diacylglycerols	1.1998	0.0830	0.3996	0.8714	0.3282	0.6229	1.1296	0.1949	0.6878	1.0455	0.1605	0.4584
PEMT Pathway													
CDP- Ethanolamine	Phospholipid Metabolism	1.2870	0.0499	0.3463	0.7928	0.0830	0.3422	1.0536	0.6454	0.9020	1.0203	0.7984	0.9052
1,2-dilinoleoyl-GPE (18:2/18:2)*	Phosphatidylethanolamines	1.0435	0.6454	0.8466	0.9911	0.8785	0.9637	1.0488	0.5737	0.8705	1.0342	0.8785	0.9420
1-oleoyl-2-arachidonoyl-GPE (18:1/20:4)*	Phosphatidylethanolamines	1.1313	0.3823	0.6968	1.0103	0.9591	0.9899	1.0933	0.2786	0.7368	1.1430	0.0830	0.3555
1-oleoyl-2-docosahexaenoyl-GPE (18:1/22:6)*	Phosphatidylethanolamines	1.0974	0.1949	0.5617	1.1017	0.1304	0.3997	1.1596	0.0650	0.5438	1.2090	0.0281	0.2184
1-oleoyl-2-linoleoyl-GPE (18:1/18:2)*	Phosphatidylethanolamines	1.0871	0.1949	0.5617	0.9944	1.0000	1.0000	1.0692	0.1949	0.6878	1.0810	0.3823	0.6591
1-palmitoyl-2-arachidonoyl-GPE (16:0/20:4)*	Phosphatidylethanolamines	0.9446	0.5737	0.8046	1.1630	0.0830	0.3422	1.0729	0.5054	0.8419	1.0986	0.3282	0.6270
1-palmitoyl-2-docosahexaenoyl-GPE (16:0/22:6)*	Phosphatidylethanolamines	1.0079	0.5737	0.8046	1.1807	0.1605	0.4420	1.0956	0.2786	0.7368	1.1900	0.1304	0.4282
1-palmitoyl-2-linoleoyl-GPE (16:0/18:2)	Phosphatidylethanolamines	0.9128	0.1304	0.4722	1.2531	0.0650	0.3072	1.0637	0.4418	0.8088	1.1439	0.3823	0.6591
1-palmitoyl-2-oleoyl-GPE (16:0/18:1)	Phosphatidylethanolamines	1.0289	0.3282	0.6636	1.1576	0.1304	0.3997	1.1754	0.1049	0.6067	1.1910	0.0650	0.3083
1-stearoyl-2-arachidonoyl-GPE (18:0/20:4)	Phosphatidylethanolamines	1.1492	0.0148	0.1782	0.9798	0.5054	0.7526	1.0848	0.0207	0.4919	1.1260	0.0030	0.0791
1-stearoyl-2-docosahexaenoyl-GPE (18:0/22:6)*	Phosphatidylethanolamines	1.0777	0.1605	0.5265	1.0594	0.0379	0.2623	1.1058	0.0499	0.5327	1.1417	0.0148	0.1573
1-stearoyl-2-linoleoyl-GPE (18:0/18:2)*	Phosphatidylethanolamines	1.1361	0.0830	0.3996	0.9913	0.8785	0.9637	1.0741	0.1605	0.6249	1.1262	0.0650	0.3083
1-stearoyl-2-oleoyl-GPE (18:0/18:1)	Phosphatidylethanolamines	1.1490	0.0207	0.2265	0.9489	0.3282	0.6229	1.1731	0.0019	0.1553	1.0902	0.0281	0.2184
Methyl Donor Pathway													
Betaine	Glycine, Serine and Threonine Metabolism	0.9805	0.7984	0.9136	0.9111	0.0207	0.1966	1.0217	0.2786	0.7368	0.8934	0.0148	0.1573
Dimethylglycine	Glycine, Serine and Threonine Metabolism	0.9282	0.7984	0.9136	1.4798	0.0047	0.1816	1.3165	0.0281	0.5264	1.3736	0.0281	0.2184
Methionine	Methionine, Cysteine, SAM and Taurine Metabolism	0.8720	0.4418	0.7331	1.0653	0.5737	0.7898	0.8634	0.3823	0.7673	0.9289	0.7209	0.8810
Cysteine	Methionine, Cysteine, SAM and Taurine Metabolism	1.0701	0.6454	0.8466	1.1522	0.0830	0.3422	1.1446	0.1304	0.6067	1.2329	0.0650	0.3083
Other Metabolites													
S-adenosylmethionine (SAM)	Methionine, Cysteine, SAM and Taurine Metabolism	1.0374	0.9591	0.9779	0.9806	0.9591	0.9899	1.0004	0.9591	0.9900	1.0172	1.0000	1.0000
S-adenosylhomocysteine (SAH)	Methionine, Cysteine, SAM and Taurine Metabolism	1.1854	0.0148	0.1782	0.9513	0.4418	0.7111	0.9318	0.3823	0.7673	1.1277	0.0499	0.2801
Serine	Glycine, Serine and Threonine Metabolism	0.7655	0.1049	0.4391	1.3165	0.0207	0.1966	1.0045	0.6454	0.9020	1.0078	1.0000	1.0000
Glycine	Glycine, Serine and Threonine Metabolism	0.9433	0.5054	0.7781	1.0195	0.5737	0.7898	0.9952	0.7209	0.9383	0.9617	0.7984	0.9052
Trimethylamine N-oxide (TMAO)	Phospholipid Metabolism	0.5548	0.0148	0.1782	1.6200	0.0207	0.1966	0.9942	0.7209	0.9383	0.8988	0.2345	0.5324
**FETAL BRAIN**													
Choline	Phospholipid Metabolism	0.9710	0.5054	0.8896	0.9404	0.2345	0.6243	1.0360	0.5737	0.8563	0.9131	0.1605	0.4224
CDP-Choline Pathway													
Phosphocholine	Phospholipid Metabolism	1.0485	0.3823	0.8075	0.9274	0.1949	0.5944	0.9793	0.8785	0.9772	0.9724	0.6454	0.8067
CDP- Choline	Phospholipid Metabolism	1.2289	0.0830	0.4626	0.6625	0.0030	0.1792	0.9731	0.6454	0.8874	0.8142	0.0650	0.2907

1,2-dilinoleoyl-GPC (18:2/18:2)	Phosphatidylcholine	1.0199	1.0000	1.0000	1.3313	0.3282	0.6597	1.0373	1.0000	1.0000	1.3490	0.3442	0.6239
1,2-dioleoyl-GPC (18:1/18:1)	Phosphatidylcholine	1.0048	0.9591	0.9904	1.0432	0.1304	0.5347	1.0334	0.6454	0.8874	1.0483	0.2786	0.5499
1,2-dipalmitoyl-GPC (16:0/16:0)	Phosphatidylcholine	1.0000	0.7209	0.9394	0.9871	0.5737	0.7897	1.0686	0.1049	0.5466	0.9872	0.7984	0.8921
1-linoleoyl-2-arachidonoyl-GPC (18:2/20:4n6)*	Phosphatidylcholine	1.0718	0.3282	0.8032	1.1954	0.7984	0.9008	1.0625	0.4418	0.7696	1.2812	0.1049	0.3477
1-myristoyl-2-arachidonoyl-GPC (14:0/20:4)*	Phosphatidylcholine	1.0575	0.6454	0.9317	1.0876	0.3823	0.6886	1.0909	0.2786	0.6542	1.1501	0.1049	0.3477
1-myristoyl-2-palmitoyl-GPC (14:0/16:0)	Phosphatidylcholine	1.0131	0.8785	0.9757	0.9915	0.6454	0.8498	1.0525	0.2786	0.6542	1.0045	0.7209	0.8465
1-oleoyl-2-docosahexaenoyl-GPC (18:1/22:6)*	Phosphatidylcholine	0.9918	1.0000	1.0000	1.0521	0.4418	0.7095	1.0415	0.4418	0.7696	1.0434	0.5054	0.7414
1-oleoyl-2-linoleoyl-GPC (18:1/18:2)*	Phosphatidylcholine	1.0308	0.9591	0.9904	1.1348	0.7209	0.8769	1.0779	0.9591	0.9995	1.1698	0.6454	0.8067
1-palmitoleoyl-2-linoleoyl-GPC (16:1/18:2)*	Phosphatidylcholine	1.0870	0.3823	0.8075	1.1546	0.3823	0.6886	1.0948	0.1605	0.5846	1.2551	0.0207	0.1833
1-palmitoyl-2-arachidonoyl-GPC (16:0/20:4n6)	Phosphatidylcholine	1.0401	0.2786	0.7488	1.0305	0.4418	0.7095	1.0480	0.2345	0.6303	1.0718	0.1304	0.3854
1-palmitoyl-2-dihomo-linolenoyl-GPC (16:0/20:3n3 or 6)*	Phosphatidylcholine	1.0264	0.5737	0.9089	1.0573	0.3282	0.6597	1.0233	0.4418	0.7696	1.0852	0.1605	0.4224
1-palmitoyl-2-docosahexaenoyl-GPC (16:0/22:6)	Phosphatidylcholine	0.9947	0.9591	0.9904	1.0900	0.0499	0.4205	1.0565	0.3282	0.6865	1.0842	0.3282	0.5967
1-palmitoyl-2-linoleoyl-GPC (16:0/18:2)	Phosphatidylcholine	1.0367	0.2345	0.6886	1.0119	0.5054	0.7556	1.0602	0.1304	0.5695	1.0490	0.0650	0.2907
1-palmitoyl-2-oleoyl-GPC (16:0/18:1)	Phosphatidylcholine	1.0238	0.8785	0.9757	1.0127	0.7209	0.8769	1.0554	0.3282	0.6865	1.0368	0.5737	0.7788
1-palmitoyl-2-stearoyl-GPC (16:0/18:0)	Phosphatidylcholine	0.9928	0.9591	0.9904	1.0522	0.1949	0.5944	1.1032	0.0379	0.5238	1.0446	0.3282	0.5967
1-stearoyl-2-arachidonoyl-GPC (18:0/20:4)	Phosphatidylcholine	1.0400	0.5054	0.8896	1.0584	0.1949	0.5944	1.0543	0.3282	0.6865	1.1008	0.0830	0.3192
1-stearoyl-2-docosahexaenoyl-GPC (18:0/22:6)	Phosphatidylcholine	1.0405	0.7209	0.9394	1.0932	0.2786	0.6406	1.0333	0.6454	0.8874	1.1375	0.0379	0.2528
1-stearoyl-2-linoleoyl-GPC (18:0/18:2)*	Phosphatidylcholine	1.0886	0.1049	0.5306	0.9933	0.7209	0.8769	1.0318	0.8785	0.9772	1.0814	0.2345	0.5079
1-stearoyl-2-oleoyl-GPC (18:0/18:1)	Phosphatidylcholine	1.0496	0.6454	0.9317	1.0723	0.4418	0.7095	1.0647	0.2345	0.6303	1.1255	0.0650	0.2907
ceramide (d18:1/14:0, d16:1/16:0)*	Ceramides	1.0341	0.7984	0.9556	1.0428	0.6454	0.8498	1.0349	0.8785	0.9772	1.0784	0.4418	0.6994
ceramide (d18:1/17:0, d17:1/18:0)*	Ceramides	0.9961	0.7984	0.9556	1.1900	0.2786	0.6406	1.1649	0.3282	0.6865	1.1853	0.2345	0.5079
ceramide (d18:2/24:1, d18:1/24:2)*	Ceramides	0.9776	0.7209	0.9394	1.1208	0.1949	0.5944	1.0834	0.2786	0.6542	1.0957	0.1049	0.3477
N-behenoyl-sphingadienine (d18:2/22:0)*	Ceramides	1.2538	0.9581	0.9904	1.7646	0.1033	0.5347	1.0821	0.4418	0.7696	2.2123	0.0650	0.2907
N-palmitoyl-sphingosine (d18:1/16:0)	Ceramides	1.0272	0.8785	0.9757	1.0508	0.5737	0.7897	1.0533	0.4418	0.7696	1.0793	0.5054	0.7414
N-stearoyl-sphingadienine (d18:2/18:0)*	Ceramides	0.9091	0.1605	0.6040	1.1501	0.1304	0.5347	1.1200	0.2345	0.6303	1.0456	0.8785	0.9296
N-stearoyl-sphingosine (d18:1/18:0)*	Ceramides	0.9545	0.5737	0.9089	1.0417	0.3282	0.6597	1.0938	0.0830	0.5466	0.9943	0.9591	0.9721
N-palmitoyl-sphinganine (d18:0/16:0)	Ceramides	1.0661	0.7209	0.9394	1.0481	0.7209	0.8769	1.0673	0.3823	0.7302	1.1173	0.1949	0.4480
N-stearoyl-sphinganine (d18:0/18:0)*	Ceramides	0.9659	0.7984	0.9556	0.9683	0.7984	0.9008	1.0535	0.5737	0.8563	0.9353	0.6454	0.8067
behenoyl sphingomyelin (d18:1/22:0)*	Sphingomyelins	1.0400	0.6454	0.9317	1.1206	0.2345	0.6243	1.0884	0.1949	0.5846	1.1654	0.0830	0.3192
lignoceroyl sphingomyelin (d18:1/24:0)	Sphingomyelins	0.9862	0.7209	0.9394	1.1093	0.3282	0.6597	1.1143	0.1949	0.5846	1.0940	0.1605	0.4224
palmitoyl sphingomyelin (d18:1/16:0)	Sphingomyelins	1.0911	0.1304	0.5771	1.0305	0.3823	0.6886	1.0505	0.5054	0.8224	1.1244	0.7209	0.8465
sphingomyelin (d18:1/14:0, d16:1/16:0)*	Sphingomyelins	1.1255	0.1304	0.5771	1.2008	0.3823	0.6886	1.1158	0.1605	0.5846	1.3514	0.0281	0.2136
sphingomyelin (d18:1/17:0, d17:1/18:0, d19:1/16:0)	Sphingomyelins	1.1459	0.2345	0.6886	1.1244	0.7209	0.8769	1.1118	0.3282	0.6865	1.2884	0.0830	0.3192
sphingomyelin (d18:1/18:1, d18:2/18:0)	Sphingomyelins	0.9816	0.8785	0.9757	1.1252	0.6454	0.8498	1.1905	0.1605	0.5846	1.1045	0.5737	0.7788
sphingomyelin (d18:1/20:0, d16:1/22:0)*	Sphingomyelins	0.9903	1.0000	1.0000	1.0723	0.4418	0.7095	1.0420	0.6454	0.8874	1.0619	0.5737	0.7788
sphingomyelin (d18:1/20:1, d18:2/20:0)*	Sphingomyelins	1.1611	0.4275	0.8580	0.8111	0.3685	0.6886	0.9190	1.0000	1.0000	0.9306	0.7923	0.8921
sphingomyelin (d18:1/22:1, d18:2/22:0, d16:1/24:1)*	Sphingomyelins	0.9921	0.9591	0.9904	1.1598	0.5737	0.7897	1.0996	0.2786	0.6542	1.1507	0.4418	0.6994
sphingomyelin (d18:1/24:1, d18:2/24:0)*	Sphingomyelins	1.0651	0.5054	0.8896	1.0888	0.7209	0.8769	1.0930	0.2345	0.6303	1.1598	0.2345	0.5079
sphingomyelin (d18:2/16:0, d18:1/16:1)*	Sphingomyelins	1.0889	0.1605	0.6040	1.2553	0.1605	0.5766	1.1192	0.1304	0.5695	1.3669	0.0499	0.2906
sphingomyelin (d18:2/24:1, d18:1/24:2)*	Sphingomyelins	0.9917	1.0000	1.0000	1.1965	0.2786	0.6406	1.0661	0.5737	0.8563	1.1866	0.3823	0.6479
stearoyl sphingomyelin (d18:1/18:0)	Sphingomyelins	0.9990	1.0000	1.0000	1.0271	0.7209	0.8769	1.1080	0.1605	0.5846	1.0261	0.6454	0.8067
palmitoyl dihydrosphingomyelin (d18:0/16:0)*	Sphingomyelins	1.1295	0.1304	0.5771	1.1080	0.3282	0.6597	1.0974	0.3823	0.7302	1.2514	0.0650	0.2907
sphingomyelin (d18:0/18:0, d19:0/17:0)*	Sphingomyelins	1.0215	0.8785	0.9757	1.1931	0.1605	0.5766	1.1542	0.1304	0.5695	1.2188	0.0830	0.3192
oleoyl-arachidonoyl-glycerol (18:1/20:4) [2]*	Diacylglycerols	1.0840	0.5054	0.8896	1.1750	0.1949	0.5944	1.2991	0.0499	0.5240	1.2737	0.0379	0.2528
palmitoyl-arachidonoyl-glycerol (16:0/20:4) [1]*	Diacylglycerols	1.1274	0.3823	0.8075	1.0821	0.7209	0.8769	1.1400	0.3823	0.7302	1.2200	0.0281	0.2136
palmitoyl-arachidonoyl-glycerol (16:0/20:4) [2]*	Diacylglycerols	1.1741	0.0379	0.3215	1.0975	0.3282	0.6597	1.2591	0.0499	0.5240	1.2885	0.0281	0.2136
palmitoyl-docosahexaenoyl-glycerol (16:0/22:6) [1]*	Diacylglycerols	1.0118	0.8785	0.9757	0.9050	1.0000	1.0000	0.8636	0.6454	0.8874	0.9157	0.7984	0.8921
palmitoyl-docosahexaenoyl-glycerol (16:0/22:6) [2]*	Diacylglycerols	1.2478	0.3717	0.8075	0.9045	0.7984	0.9008	1.1577	0.3717	0.7302	1.1286	0.4945	0.7414
stearoyl-arachidonoyl-glycerol (18:0/20:4) [1]*	Diacylglycerols	0.9656	0.7984	0.9556	1.1864	0.1949	0.5944	1.0351	0.7209	0.9229	1.1456	0.1949	0.4480
stearoyl-arachidonoyl-glycerol (18:0/20:4) [2]*	Diacylglycerols	1.0776	0.6454	0.9317	1.1608	0.1949	0.5944	1.1268	0.5737	0.8563	1.2509	0.0499	0.2906
stearoyl-docosahexaenoyl-glycerol (18:0/22:6) [1]*	Diacylglycerols	1.0781	0.8785	0.9757	0.9966	0.8785	0.9455	1.0493	0.8785	0.9772	1.0744	0.7209	0.8465
stearoyl-docosahexaenoyl-glycerol (18:0/22:6) [2]*	Diacylglycerols	1.1247	0.7209	0.9394	0.9786	0.7984	0.9008	1.1389	0.1304	0.5695	1.1007	0.2345	0.5079
PEMT Pathway													
CDP- Ethanolamine	Phospholipid Metabolism	1.0831	0.0104	0.1529	0.8813	0.0011	0.0825	1.0060	0.9591	0.9995	0.9546	0.0650	0.2907
1-linoleoyl-2-arachidonoyl-GPE (18:2/20:4)*	Phosphatidylethanolamines	1.1450	0.4418	0.8580	1.0536	1.0000	1.0000	0.9891	0.8785	0.9772	1.2064	0.3823	0.6479
1-oleoyl-2-arachidonoyl-GPE (18:1/20:4)*	Phosphatidylethanolamines	0.9976	0.9591	0.9904	1.0352	0.1949	0.5944	1.0170	0.7209	0.9229	1.0328	0.6454	0.8067
1-oleoyl-2-docosahexaenoyl-GPE (18:1/22:6)*	Phosphatidylethanolamines	0.9914	0.5054	0.8896	1.0115	0.6454	0.8498	0.9989	0.7984	0.9524	1.0028	0.3823	0.6479
1-oleoyl-2-linoleoyl-GPE (18:1/18:2)*	Phosphatidylethanolamines	1.1711	0.5054	0.8896	1.3260	0.5054	0.7556	0.6839	0.4005	0.7600	1.5528	0.1949	0.4480
1-palmitoyl-2-arachidonoyl-GPE (16:0/20:4)*	Phosphatidylethanolamines	1.0097	0.7209	0.9394	1.0475	0.2345	0.6243	1.0292	0.5054	0.8224	1.0577	0.1605	0.4224
1-palmitoyl-2-docosahexaenoyl-GPE (16:0/22:6)*	Phosphatidylethanolamines	1.0351	0.6454	0.9317	1.0057	0.9591	0.9884	1.0694	0.3823	0.7302	1.0410	0.9591	0.9721
1-palmitoyl-2-oleoyl-GPE (16:0/18:1)	Phosphatidylethanolamines	1.0170	0.5737	0.9089	1.0571	0.2786	0.6406	1.0592	0.1949	0.5846	1.0750	0.1605	0.4224
1-palmitoyl-2-stearoyl-GPE (16:0/18:0)*	Phosphatidylethanolamines	1.0231	0.5737	0.9089	1.0399	0.3823	0.6886	1.0940	0.1049	0.5466	1.0640	0.2345	0.5079
1-stearoyl-2-arachidonoyl-GPE (18:0/20:4)	Phosphatidylethanolamines	1.0032	0.8785	0.9757	1.0574	0.1304	0.5347	1.0649	0.0650	0.5240	1.0608	0.1049	0.3477
1-stearoyl-2-docosahexaenoyl-GPE (18:0/22:6)*	Phosphatidylethanolamines	1.0194	1.0000	1.0000	1.0329	0.8785	0.9455	1.0845	0.2345	0.6303	1.0529	0.6454	0.8067
1-stearoyl-2-oleoyl-GPE (18:0/18:1)	Phosphatidylethanolamines	1.0674	0.4418	0.8580	1.0702	0.3282	0.6597	1.0774	0.1304	0.5695	1.1424	0.0650	0.2907
Methyl Donor Pathway													
Betaine	Glycine, Serine and Threonine Metabolism	1.0139	0.8785	0.9757	1.0471	0.7984	0.9008	0.9386	0.8785	0.9772	1.0616	0.7209	0.8465
Dimethylglycine	Glycine, Serine and Threonine Metabolism	0.7921	0.0650	0.4137	1.4138	0.0047	0.1887	1.1206	0.2345	0.6303	1.1199	0.3282	0.5967
Sarcosine	Glycine, Serine and Threonine Metabolism	1.0518	0.7209	0.9394	1.5091	0.0207	0.3217	0.7751	0.1605	0.5846	1.5872	0.0104	0.1358
Methionine	Methionine, Cysteine, SAM and Taurine Metabolism	0.8954	0.8785	0.9757	0.8438	0.0650	0.4355	0.7393	0.0104	0.3865	0.7555	0.0104	0.1358
Cysteine	Methionine, Cysteine, SAM and Taurine Metabolism	1.3784	0.0207	0.2145	0.8161	0.0379	0.3968	1.1482	0.3282	0.6865	1.1249	0.3823	0.6479
Other Metabolites													
S-adenosylmethionine (SAM)	Methionine, Cysteine, SAM and Taurine Metabolism	1.0412	0.3282	0.8032	0.9132	0.0148	0.3090	0.9828	0.9591	0.9995	0.9509	0.3282	0.5967
S-adenosylhomocysteine (SAH)	Methionine, Cysteine, SAM and Taurine Metabolism	0.9381	0.6454	0.9317	0.9678	0.7984	0.9008	0.8640	0.1949	0.5846	0.9079	0.2786	0.5499
Serine	Glycine, Serine and Threonine Metabolism	0.7988	0.0047	0.0936	1.0714	0.3282	0.6597	1.0245	0.5737	0.8563	0.8558	0.0148	0.1671
Glycine	Glycine, Serine and Threonine Metabolism	0.9305	0.3282	0.8032	1.0583	0.3282	0.6597	1.0987	0.1304	0.5695	0.9847	0.8785	0.9296
Trimethylamine N-oxide (TMAO)	Phospholipid Metabolism	0.7854	0.1049	0.5306	1.2773	0.0650	0.4355	1.0442	1.0000	1.0000	1.0032	1.0000	1.0000
