# Supplementary material for: Alcohol Exposure May Increase Prenatal Choline Needs Through Redirection of Choline into Lipid Synthesis Rather than Methyl Donation
Source: Metabolites. 2025 Apr 24;15(5):289. doi: 10.3390/metabo15050289 (PMC12113322; doi:10.3390/metabo15050289)
Supplement: Supplementary file 1 [file metabolites-15-00289-s001.zip › Supplemental Table 2.pdf]

Table S2. Choline-Related Lipid Metabolites and Amount Changed By at Least 10%

Tissue	Subpathway	ALC/CON		ALC-Cho/ALC		CON-Cho/CON		ALC-Cho/CON	
		Number of Metabolites Detected	Effect	Number of Metabolites Detected	Effect	Number of Metabolites Detected	Effect	Number of Metabolites Detected	Effect
Maternal Plasma	Phosphatidylcholines	20	13 increased, 4 decreased	20	1 increased,7 decreased	20	9 increased, 4 decreased	20	12 increased, 4 decreased
	Sphingomyelins	23	18 increased	23	5 decreased	23	22 increased	23	16 increased
	Diacylglycerols	20	10 increased, 4 decreased	20	6 increased, 10 decreased	20	8 increased, 3 decreased	20	10 increased, 5 decreased
	Ceramides	4	3 increased, 1 decreased	4	1 increased	4	3 increased, 1 decreased	4	1 increased, 1 decreased
	Phosphatidylethanolamines	13	4 increased, 6 decreased	13	3 increased, 2 decreased	13	4 increased, 7 decreased	13	6 increased, 7 decreased
Maternal Liver	Phosphatidylcholines	22	14 increased, 1 decreased	22	2 increased, 6 decreased	22	5 increased, 5 decreased	22	11 increased, 1 decreased
	Sphingomyelins	21	13 increased, 1 decreased	21	10 decreased	21	12 decreased	21	9 increased, 3 decreased
	Diacylglycerols	25	15 increased, 3 decreased	25	11 increased, 3 decreased	25	14 increased, 5 decreased	25	19 increased, 3 decreased
	Ceramides	7	7 increased	7	1 increased	7	1 decreased	7	6 increased
	Phosphatidylethanolamines	13	12 increased	13	11 decreased	13	3 decreased	13	8 increased, 1 decreased
Placenta	Phosphatidylcholines	19	13 increased	19	2 decreased	19	9 increased, 1 decreased	19	7 increased, 1 decreased
	Sphingomyelins	28	19 increased, 1 decreased	28	6 increased, 4 decreased	28	20 increased, 1 decreased	28	14 increased, 1 decreased
	Diacylglycerols	20	11 increased	20	1 increased, 2 decreased	20	11 increased	20	11 increased, 1 decreased
	Ceramides	8	3 increased	8	1 increased, 4 decreased	8	4 increased	8	1 increased, 1 decreased
	Phosphatidylethanolamines	12	4 increased	12	5 increased	12	4 increased	12	8 increased
Fetal Brain	Phosphatidylcholines	19		19	4 increased	19	1 increased	19	8 increased
	Sphingomyelins	15	4 increased	15	10 increased, 1 decreased	15	7 increased	15	11 increased
	Diacylglycerols	9	4 increased	9	3 increased	9	6 increased, 1 decreased	9	7 increased
	Ceramides	9	1 increased	9	4 increased	9	2 increased	9	3 increased
	Phosphatidylethanolamines	11	2 increased	11	1 increased	11	1 decreased	11	3 increased
