# Supplementary material for: Alcohol Exposure May Increase Prenatal Choline Needs Through Redirection of Choline into Lipid Synthesis Rather than Methyl Donation
Source: Metabolites. 2025 Apr 24;15(5):289. doi: 10.3390/metabo15050289 (PMC12113322; doi:10.3390/metabo15050289)
Supplement: Supplementary file 1 [file metabolites-15-00289-s001.zip › Supplemental.pdf]

Table S3. Fold-Change (Alcohol + Choline /Control) in Choline-Related Metabolites in Maternal-Fetal Tissues.

Metabolites	Maternal Plasma	Maternal Liver	Placenta	Fetal Brain
**Choline**	0.9953	**1.3796***	1.0356	0.9131
		**CDP- Choline Pathway**		
**Phosphocholine**	0.6089 (L)	**1.7084***	1.0518	0.9724
**CDP- Choline**	ND	1.3742 (L)	0.9437	0.8142 (L)
**Phosphatidylcholines**	1.1632 (L)	1.0428	**1.0680# (L)**	1.0485
**Ceramides**	**1.4072# (L)**	1.4335 (L)	1.0034	1.0271
**Sphingomyelins**	1.1992 (L)	1.0091	1.0568	1.1252 (M)
**Diacylglycerols**	0.9864	1.1508 (S)	1.0403	1.1966 (L)
		**PEMT Pathway**		
**CDP- Ethanolamine**	ND	0.9220	1.0203	0.9546
**Phosphatidylethanolamines**	1.0912	1.1634 (M)	**1.1356***	1.0659
**SAM**	ND	0.9219	1.0172	0.9509
**SAH**	0.6965 (L)	1.1028 (M)	1.1277 (L)	0.9079
**SAM/SAH Ratio**	ND	0.8324 (L)	0.8794 (M)	1.0516
		**Methyl Donor Pathway**		
**Betaine**	1.0802	1.5968 (L)	0.8934 (L)	1.0616
**Dimethylglycine**	**1.3223# (L)**	1.2103 (L)	1.3736 (L)	1.1199 (M)
**Sarcosine**	ND	1.5010 (S)	ND	1.5872 (L)
**Methionine**	0.8513 (S)	0.8999 (S)	0.9289	0.7555 (L)
**Cysteine**	1.0918	1.0312	1.2329 (L)	1.1249 (S)
		**Other Metabolites**		
**Serine**	0.9340	1.0311	1.0078	0.8558 (L)
**Glycine**	0.8115 (S)	1.1116 (S)	0.9617	0.9847
**Serine/Glycine Ratio**	1.0748	0.9376	1.0509	0.8781 (L)
**TMAO**	0.9179	0.8817 (S)	0.8988 (M)	1.0032

* FDR < 0.05, # 0.05 < FDR < 0.1. FDR - False Discovery Rate; (L) – large effect size; (M) – medium effect size; (S) – small effect size; ND - Not Detected; CDP- cytidine diphosphate; PEMT - Phosphatidylethanolamine N-Methyltransferase; TMAO- Trimethylamine N-oxide; SAM – S-Adenosyl Methionine; SAH – S-Adenosyl Homocysteine

Table S4. Fold-Change (Control + Choline /Control) in Choline-Related Metabolites in Maternal-Fetal Tissues.

Metabolites	Maternal Plasma	Maternal Liver	Placenta	Fetal Brain
**Choline**	1.0931	1.0937	0.9713	1.0360
**CDP- Choline Pathway**				
**Phosphocholine**	1.0124	1.6480 (L)	1.0204	0.9793
**CDP- Choline**	ND	1.4968 (L)	1.0048	0.9731
**Phosphatidylcholines**	1.1408 (M)	0.9786	**1.0950***	1.0598
**Ceramides**	1.2635 (S)	0.9819	1.0878	1.0898
**Sphingomyelins**	1.3246 (M)	0.8826 (S)	**1.0835# (L)**	1.0857
**Diacylglycerols**	1.0013	1.0516	1.0793	1.1268 (M)
**PEMT Pathway**				
**CDP- Ethanolamine**	ND	1.1855 (L)	1.0536	1.0060
**Phosphatidylethanolamines**	1.0366	0.9680	**1.0975# (L)**	1.0571
**SAM**	ND	0.9716	1.0004	0.9828
**SAH**	1.6805 (S)	0.9501	0.9318	0.8640 (M)
**SAM/SAH Ratio**	ND	0.9783	1.0653	1.1368 (M)
**Methyl Donor Pathway**				
**Betaine**	1.2742 (L)	1.4376 (L)	1.0217	0.9386
**Dimethylglycine**	1.2576 (L)	1.1436 (M)	1.3165 (L)	1.1206 (M)
**Sarcosine**	ND	1.2436 (M)	ND	0.7751 (M)
**Methionine**	0.8410 (M)	0.7627 (M)	0.8634 (S)	0.7393 (L)
**Cysteine**	1.3709 (L)	1.0671	1.1446 (M)	1.1482 (M)
**Other Metabolites**				
**Serine**	1.0585	0.9190	1.0045	1.0245
**Glycine**	0.8486 (S)	1.0114	0.9952	1.0987
**Serine/Glycine Ratio**	1.1129 (S)	0.9197	1.0127	0.9477
**TMAO**	0.9301	0.5909 (M)	0.9942	1.0442

* FDR < 0.05, # 0.05 < FDR < 0.1. FDR - False Discovery Rate; (L) – large effect size; (M) – medium effect size; (S) – small effect size; ND - Not Detected; CDP- cytidine diphosphate; PEMT - Phosphatidylethanolamine N-Methyltransferase; TMAO- Trimethylamine N-oxide; SAM – S-Adenosyl Methionine; SAH – S-Adenosyl Homocysteine

Table S5. Gestational and Fetal Weight Outcomes

	CON	CON-Cho	ALC	ALC- Cho
**Gestational Weight Gain (E0.5 – E17.5) (g)**	12.89 ± 1.79	15.52 ± 1.87	10.47 ± 1.46	12.68 ± 1.78
**Gavage Weight Gain (E8.5 – E17.5) (g)**	10.83 ± 2.00	12.49 ± 1.43	9.03 ± 1.29	10.48 ± 1.89
**Placenta Weight (g)**	0.091 ± 0.013	0.085 ± 0.0056	0.086 ± 0.0055	0.084 ± 0.0032
**Placental Efficiency**	10.51 ± 2.05	11.51 ± 1.54	10.14 ± 0.51	11.83 ± 2.13
**Fetal Body Weight (g)**	0.93 ± 0.12	0.97 ± 0.11	0.87 ± 0.076	0.98 ± 0.17
**Fetal Brain Weight (g)**	0.063 ± 0.0051	0.060 ± 0.0076	0.059 ± 0.0036	0.056 ± 0.0068

All values represented as mean ± SD. ALC – Alcohol-exposed; Con - Control; Cho – Choline-treated; E = Embryonic day; g = gram

A.

Comparison	Maternal Plasma	Maternal Liver	Placenta	Fetal Brain
ALC VS CON	ND	0.9062	0.7833 (L)	0.9932
ALC- Cho vs ALC	ND	1.0568	1.2978 (L)	1.0357
ALC-CHO vs CON	ND	0.9577	1.0166	1.0286
CON-Cho vs CON	ND	0.5411 (L)	0.9426	0.9210

B.

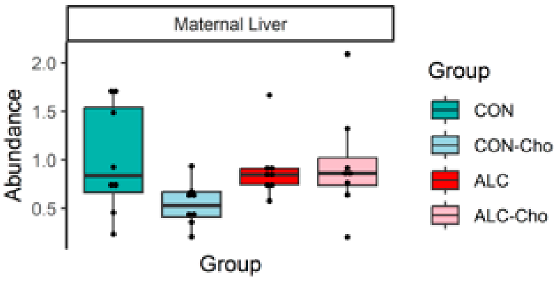

C.

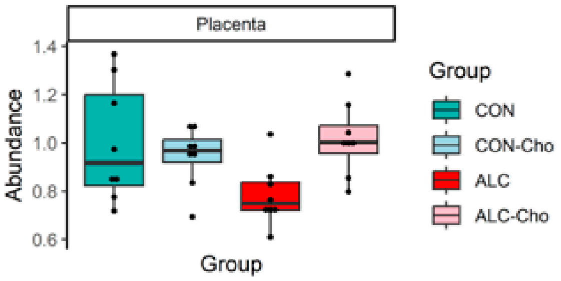

D.

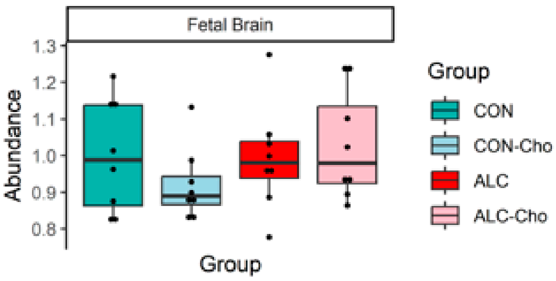

E.

Metabolites	Gestational Weight Gain	Gavage Weight Gain	Placenta Weight	Placental Efficiency	Fetal Body Weight	Fetal Brain Weight
**Maternal Liver 5MeTHF**	-0.2643	**-0.3706***	0.2386	-0.0946	0.1609	0.1924
**Placenta 5MeTHF**	**0.4084***	**0.3596***	-0.2830	0.1917	0.0667	0.0411
**Fetal Brain 5MeTHF**	-0.01173	-0.0623	-0.1884	-0.1822	-0.2397	**-0.3424#**

Figure S1 Change in 5-methyltetrahydrofolate (5MeTHF)- (A) Fold-change (FC) of 5MeTHF in all tissues (B) FC of 5 MeTHF relative to Control (CON) in maternal liver (C) placenta (D) fetal brain (E) Spearman correlation coefficient between 5MeTHF in each tissue and pregnancy outcomes ; (L) – large effect size; (M) – medium effect size; ND - Not Detected * $p < 0.05$, 0.05 < # < 0.1; CDP

A.

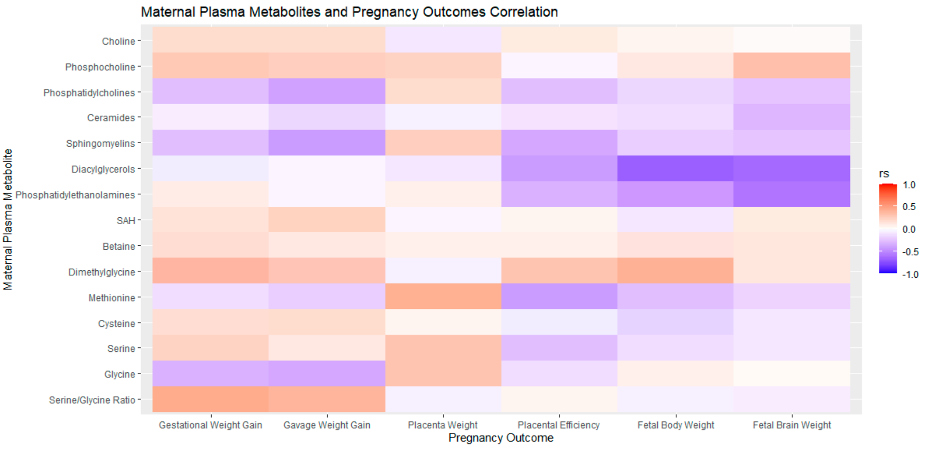

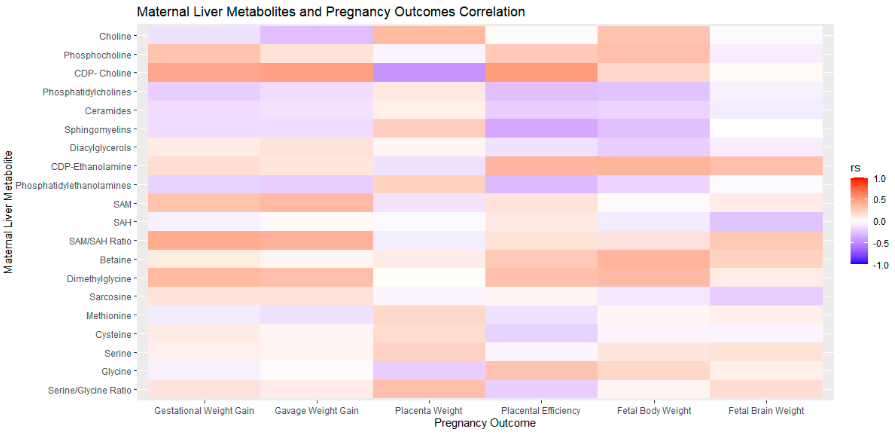

B.

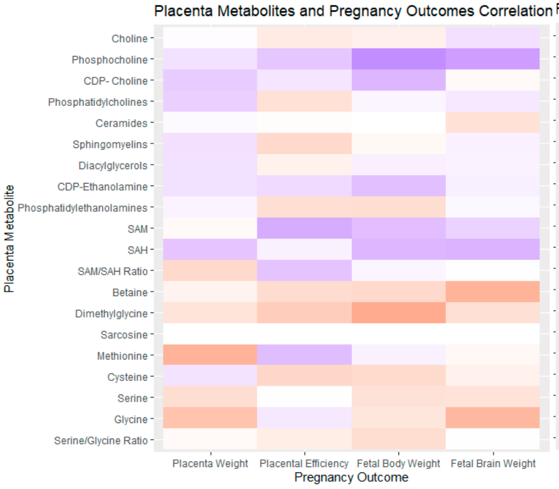

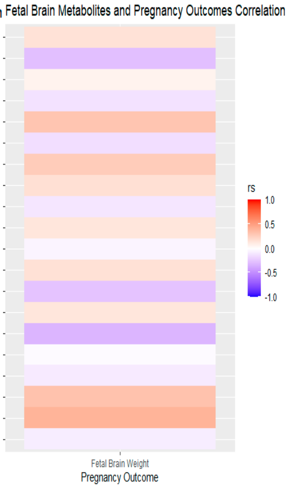

C.

Figure S2 Choline-Related Metabolites Correlated with Pregnancy Outcomes- Spearman correlation coefficient (rs) between pregnancy outcomes and choline-related metabolites in the (A) maternal plasma (B) maternal liver (C) placenta (D) fetal brain.

Figure S3. Maternal Plasma Choline-Related Metabolites Abundance Relative to Control

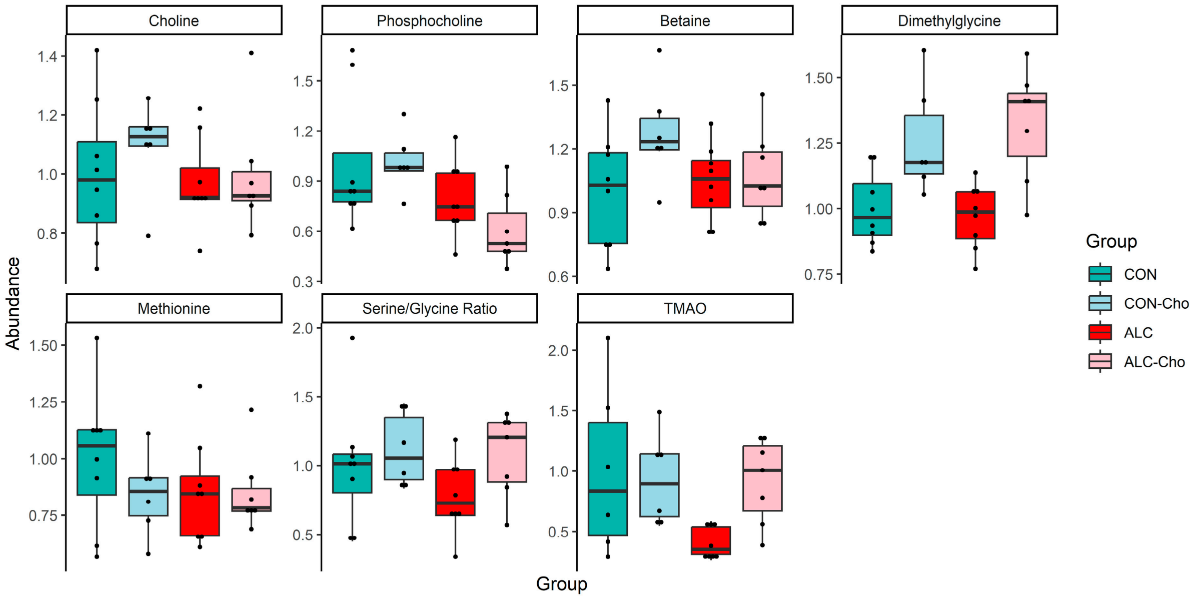

TMAO- Trimethylamine N-oxide; CON – Control; CON- Cho – Control + Choline; ALC – Alcohol; ALC- Cho – Alcohol + Choline

Figure S4. Maternal Liver Choline-Related Metabolites Abundance Relative to Control

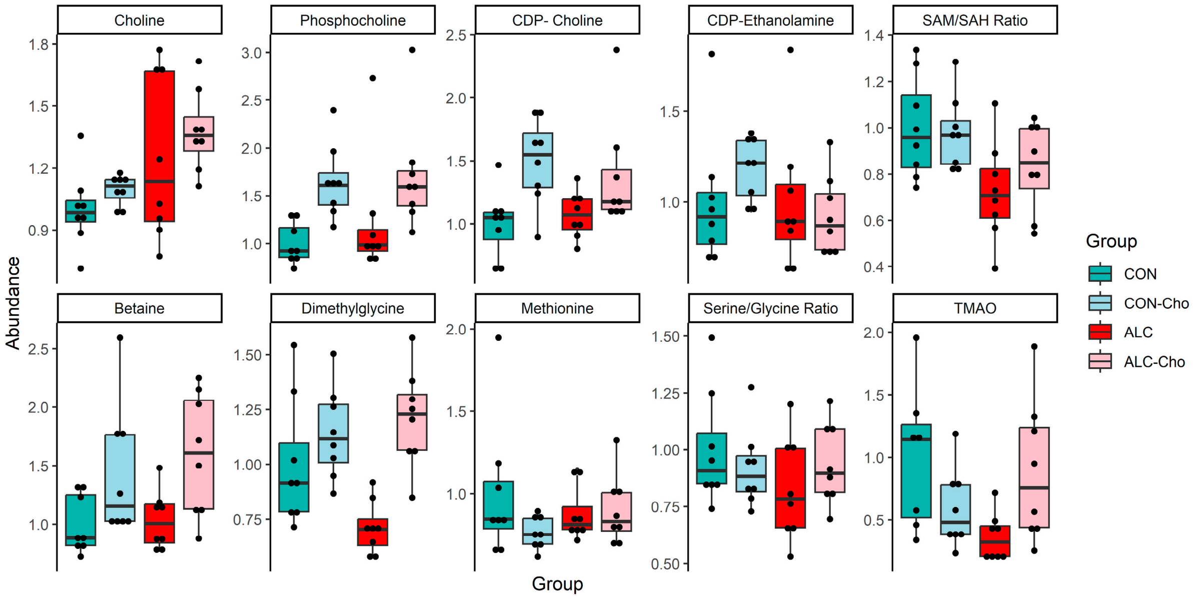

CDP- cytidine diphosphate; TMAO- Trimethylamine N-oxide; SAM – S-Adenosyl Methionine; SAH – S-Adenosyl Homocysteine; CON – Control; CON- Cho – Control + Choline; ALC – Alcohol; ALC- Cho – Alcohol + Choline

Figure S5. Placenta Choline-Related Metabolites Abundance Relative to Control

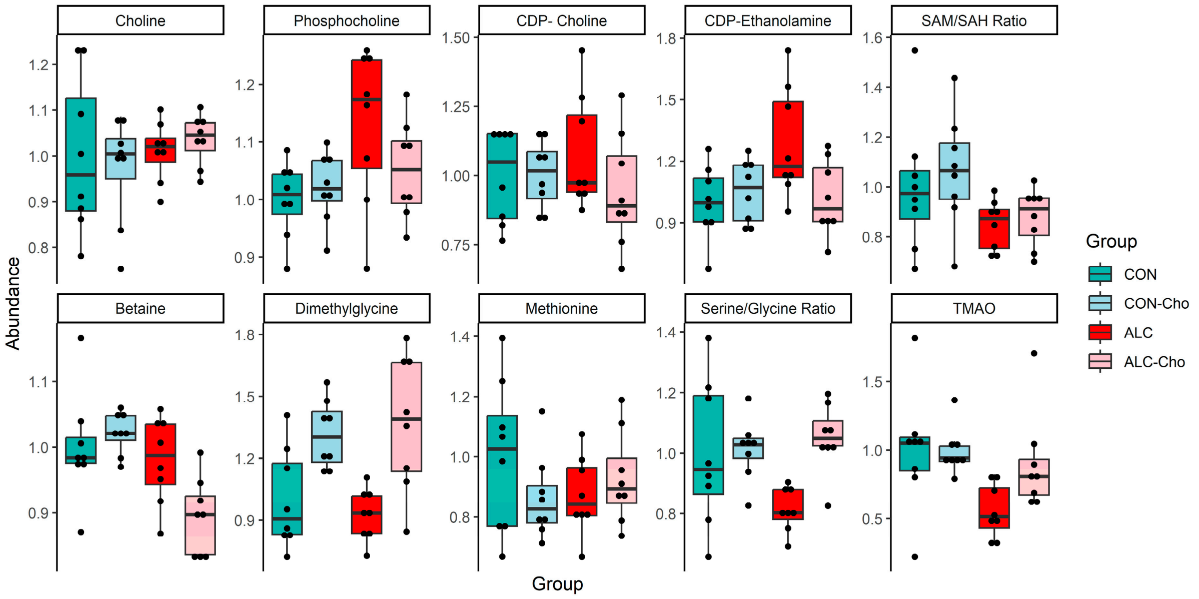

CDP- cytidine diphosphate; TMAO- Trimethylamine N-oxide; SAM – S-Adenosyl Methionine; SAH – S-Adenosyl Homocysteine; CON – Control; CON- Cho – Control + Choline; ALC – Alcohol; ALC- Cho – Alcohol + Choline

Figure S6. Fetal Brain Choline-Related Metabolites Abundance Relative to Control

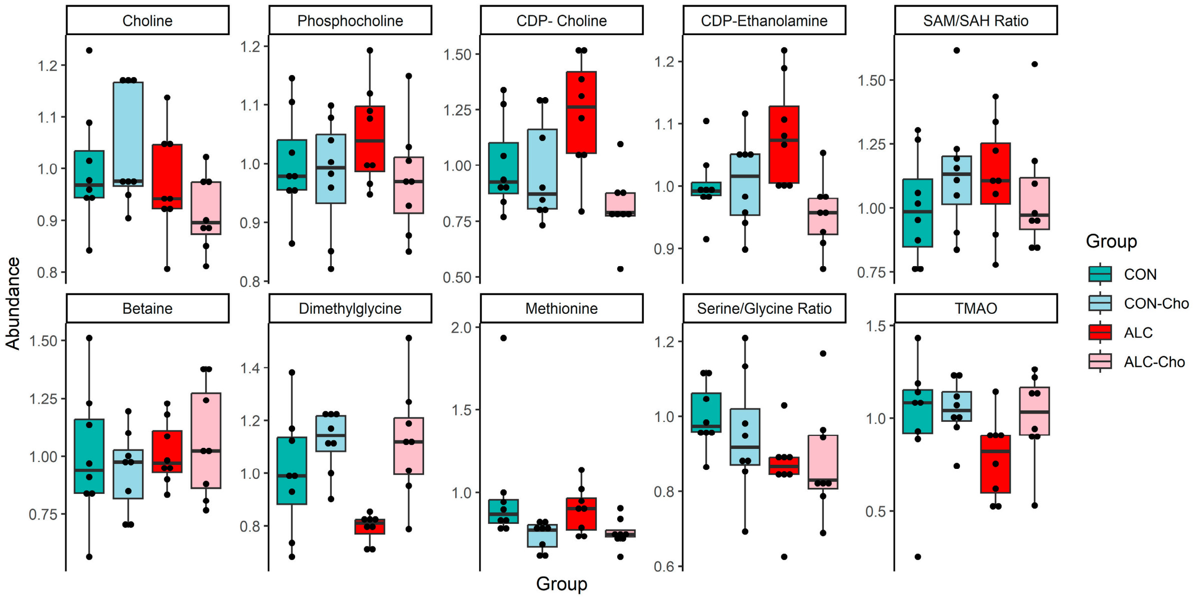

CDP- cytidine diphosphate; TMAO- Trimethylamine N-oxide; SAM – S-Adenosyl Methionine; SAH – S-Adenosyl Homocysteine; CON – Control; CON- Cho – Control + Choline; ALC – Alcohol; ALC- Cho – Alcohol + Choline
